# Identification, characterization, and prognosis investigation of pivotal genes shared in different stages of breast cancer

**DOI:** 10.1038/s41598-023-35318-x

**Published:** 2023-05-25

**Authors:** Foad Rommasi

**Affiliations:** Department of Biochemistry, Faculty of Biological Sciences, Tarbiat Modares University, Tehran, Iran

**Keywords:** Gene expression, Medical genetics, Breast cancer

## Abstract

One of the leading causes of death (20.1 per 100,000 women per year), breast cancer is the most prevalent cancer in females. Statistically, 95% of breast cancer are categorized as adenocarcinomas, and 55% of all patients may go into invasive phases; however, it can be successfully treated in approximately 70–80% of cases if diagnosed in the nascent stages. The emergence of breast tumor cells which are intensely resistant to conventional therapies, along with the high rate of metastasis occurrence, has highlighted the importance of finding novel strategies and treatments. One of the most advantageous schemes to alleviate this complication is to identify the common differentially expressed genes (DEGs) among primary and metastatic cancerous cells to use resultants for designing new therapeutic agents which are able to target both primary and metastatic breast tumor cells. In this study, the gene expression dataset with accession number GSE55715 was analyzed containing two primary tumor samples, three bone-metastatic samples, and three normal samples to distinguish the up- and down regulated genes in each stage compared to normal cells as control. In the next step, the common upregulated genes between the two experimental groups were detected by Venny online tool. Moreover, gene ontology, functions and pathways, gene-targeting microRNA, and influential metabolites were determined using EnrichR 2021 GO, KEGG pathways miRTarbase 2017, and HMDB 2021, respectively. Furthermore, elicited from STRING protein–protein interaction networks were imported to Cytoscape software to identify the hub genes. Then, identified hub genes were checked to validate the study using oncological databases. The results of the present article disclosed 1263 critical common DEGs (573 upregulated + 690 downregulated), including 35 hub genes that can be broadly used as new targets for cancer treatment and as biomarkers for cancer detection by evaluation of expression level. Besides, this study opens a new horizon to reveal unknown aspects of cancer signaling pathways by providing raw data evoked from in silico experiments. This study’s outcomes can also be widely utilized in further lab research since it contains diverse information on common DEGs of varied stages and metastases of breast cancer, their functions, structures, interactions, and associations.

## Introduction

As a complicated disease in which uncontrolled, abnormal, and rapid growth of mutated cells cause tissues and organs dysfunctions^1^, cancer should not be counted as a singular, one-facet disease but as an amalgam of more than 100 various diseases^2^. Cancer can be examined from different perspectives with a gamut from immunosurveillance^3^ to epigenetic studies^4^. The immunological survey has demonstrated that both adaptive and innate immune systems, particularly lymphocytes, have a critical role in cancer incidence. The in vivo genomic studies have also proved that the lack of some important genes such as recombinase activating gene (RAG)-2 can diminish the lymphocytes’ capacity to combat malignant cells^5^. That’s because this gene plays an influential role in the production of peripheral αβ T cells, B cells, and Natural Killer (NK) cells^6^.

**Figure 1:**
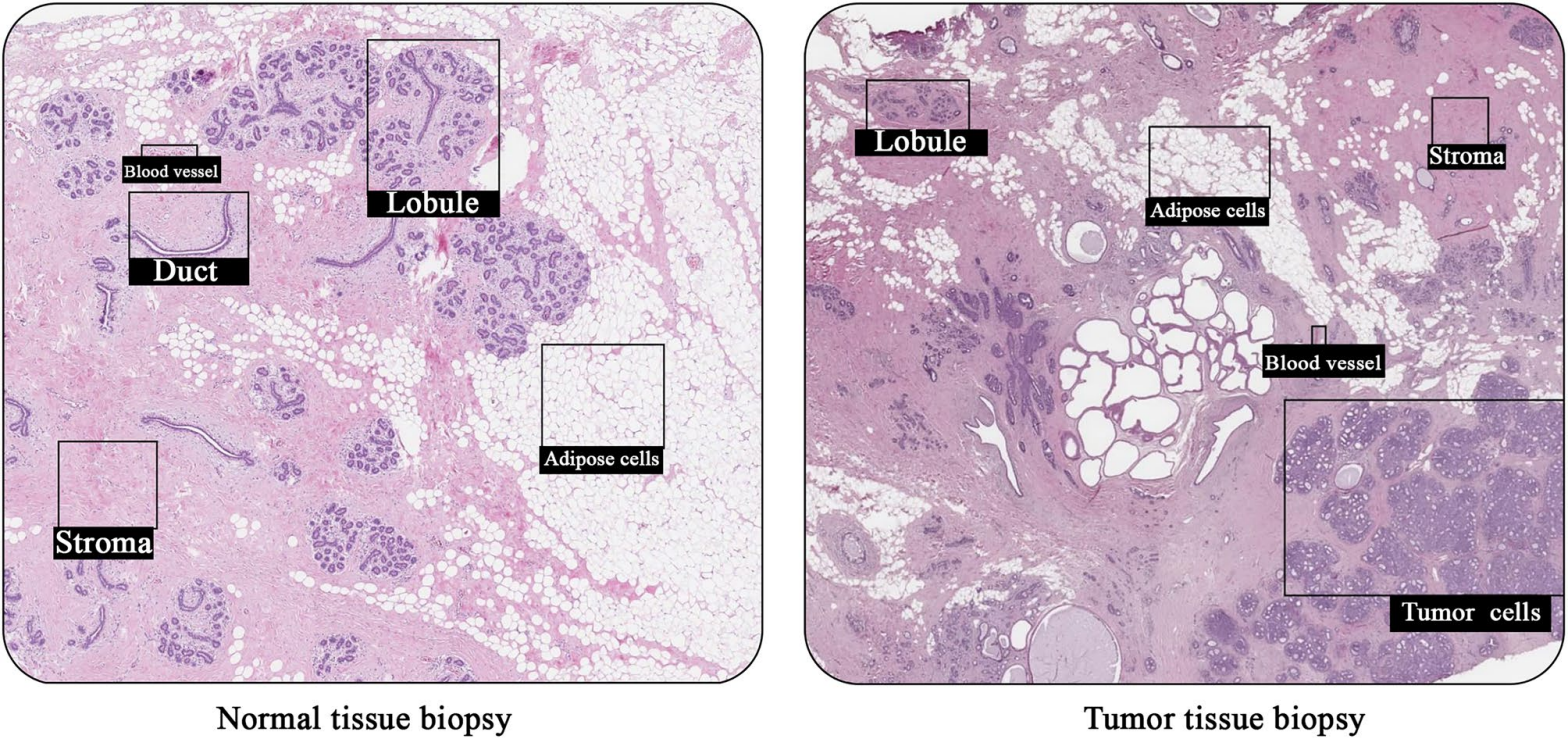
Comparison of healthy breast biopsy with a cancerous mammary biopsy (samples from a 68-year-old female with ductal carcinoma grade 1, Elston-Ellis score 4): While malignant cells can be explicitly observed in the tumor tissue biopsy, there is no cancerous cell in normal breast. Obtained from: The Human Protein Atlas (https://www.proteinatlas.org/learn/dictionary/pathology/breast+cancer#Breast-cancer-1,-ductal-carcinoma)^9,10^.

Mutations and, consequently, cancerous cells’ incidents can occur in different human tissues and organs. Breast cancer is the most common malignancy in females; however, it can be successfully treated in ~ 70–80% of patients if diagnosed timely^7^. Breast cancer or mammary carcinoma is initially caused by the uncontrolled growth of abnormal cells in the epithelium of ducts (85%) or lobules (15%)^8^. Figure 1 illustrates and compares the cancerous and normal breast tissue simultaneously.

As aforesaid, it has been reported that 95% of breast malignancies are adenocarcinomas^11^, and 55% of cases of breast cancer are diagnosed with invasive ductal carcinoma (IDC)^12^. Various symptoms have been counted as mammary carcinoma signs, but admittedly breast lump is the most prevalent symptom in patients diagnosed with breast cancer^13^. Both short and long diagnostic intervals are recorded in women with breast cancer^14^, nonetheless, evidence indicates that most patients usually experience short diagnostic intervals^15^. Long diagnostic intervals are the most concerning cases, whereas it is testified that they are associated with lower than five-year survival rates in patients^16^. Various treatment strategies are currently adopted for breast cancer, including the removal of tumors and their adjacent lymph nodes by surgical resection^17^, chemotherapy^18^, radiotherapy (which is typically associated with numerous complications)^19^, and immunotherapy. Chemotherapy agents such as Ribociclib, a cyclin-dependent kinase 4 and 6 (*CDK4/6*) inhibitor, are shown to be effective in breast cancer treatment and improving the survival rate. In a study by Seock-Ah Im et al*.*^20^, the Ribociclib treatment group had a higher survival rate than that of the placebo group (75.2% vs. 61.7%, respectively). Such studies denote the importance of detecting the contributing genes to breast cancer and utilizing appropriate therapeutic agents for the disease treatment^20^. Genetic studies have revealed the most critical anti-oncogenes and oncogenes that are related to breast cancer^21^. For instance, breast cancer-associated genes 1 and 2 (*BRCA 1* and *BRCA 2*), located on chromosomes 17q21 and 13q12 sequentially, are the coding genes that produce anti-tumor proteins suppressing malignant cells in breast tissue^22,23^. Human epidermal growth factor receptor 2 (*HER2*), located on chromosome 17q12, has also been recognized as a primary oncogene that stimulates cancer signaling pathways in cells by binding to family members of epithelial growth factor receptor (*EGFR*)^7^. Once again, these findings highlight the significance of identifying the genes which are mainly pertaining to breast cancer incidents.

There are currently various bioinformatics approaches, such as RNA-seq^24^ and Microarray^25^, to investigate the transcriptomes and analyze the gene expression level in different tissues and diseases. Such approaches are utilized to compose large quantities of gene expression analysis to simplify the detection of critical DEGs, pathways, and biomarkers correlated with a particular disease^26^. Microarray analysis can be performed using online databases such as National Center for Biotechnology Information (NCBI), Gene Expression Omnibus (GEO), and the outcomes can be downloaded as text files freely^27^. The GEO2R tool provides an opportunity to define and compare the results of high-throughput genomic data; hence, this database has been adopted to evaluate the genes’ expression in the current study.

In the present study, the gene expression dataset GSE55715, in which the gene expression of three various states, including healthy mammary epithelial cells, primary tumor cells from the right breast, and P7731 cell line (ER-/PR-/HER2-; triple negative bone metastasis breast cancer cell line) was characterized, is used for detecting the upregulated and downregulated genes in different breast cancer stages in the first step. In the second step, after exposing two cut-offs, the common DEGs in primary and metastatic tumor cells were detected using Venny to investigate these common gene profiles. Other online bioinformatics databases were also utilized to explore the TFs, miRNAs, and metabolites which are associated with such genes. Upstream and downstream regulatory pathways, along with protein–protein interactions, were identified for the protein encoding genes. The importance of this study is due to the detection of common up- and downregulated genes in both primary and metastatic cancerous cells; hence, the results can be adapted for other clinical and experimental conductions like drug designing by docking tools or pharmacological studies.

## Materials and methods

### Gene profile and microarray data

A comprehensive review was conducted to find a proper study that has suitably analyzed the transcriptome data for normal control cells, primary tumor cells, and metastatic cancerous cells. A study by Dilara Savci-Heijink et al*.*^28^, which has the microarray analysis of all desired states, was chosen for further consideration^28^. This study has the gene expression dataset with accession number GSE55715 analyzing the transcriptome pattern of 8 samples, including three samples of normal mammary epithelial cells, two samples of primary tumor cells, and three samples of the P7731 cell line. GSE55715 was obtained from the NCBI database’s GEO (http://www.ncbi.nlm.nih.gov/geo/). The platform used for array data in this analysis was the GPL6947 (Illumina HumanHT-12 V3.0 expression beadchip), and it contained eight samples, as previously mentioned^28^.

### Identification of differentially expressed genes

After selecting the appropriate study, the GEO online analyzer (http://www.ncbi.nlm.nih.gov/geo/geo2r/) was adopted to analyze the genes’ profiles and to detect DEGs^29^. GEO2R, as an online bioinformatics analyzer, allows the users to compare two or more defined groups in terms of gene expression. In order to distinguish the up- and downregulated genes in primary and tumor cells in comparison to normal breast cells, three certain groups were defined and two various analyses were implemented. In the first analysis, the gene expression pattern of 2 samples of primary tumor cells (as a first test group) was compared with the normal mammary epithelial cells (as the control group). In the next round, the expression profile of 3 metastatic tumor cells (as the second test group) was analyzed to be compared to the control group. It is also plausible to detect the genes which have a pivotal role in driving breast cancer from primary tumor to metastatic stage by doing a GEO2R transcriptome analysis where the metastatic group is the test group while the primary group is defined as the control. Zhao et al*.*^30^ conducted such analysis on three various GSE datasets to identify these genes for early detection of bone metastasis.

The cut-off conditions for detecting upregulated and downregulated genes were defined according to univariate tests. Adjusted *p* value ≤ 0.05 and |Log fold-change (Fc)|≥ 2 were defined as cut-off criteria, respectively. After applying the cut-off criteria to determine the DEGs, specific symbols of upregulated and downregulated genes were elicited using their gene code. Repetitive/duplicate genes were then deleted, and others were utilized for further analysis.

### Recognition of common up- and downregulated genes

A number of 889 and 1131 genes were detected as upregulated and downregulated genes in primary and metastatic cancerous cells compared to normal cells, respectively. In order to determine the number of common upregulated genes between these two types of cancerous cells, the Venny online tool (https://bioinfogp.cnb.csic.es/tools/venny/) was used^31^. The same strategy was adopted to recognize common downregulated genes among 946 and 1180 downregulated genes of the primary tumor and bone-metastatic cells sequentially. In the end, 573 and 690 genes were ascertained as common up- and downregulated genes sequentially. The further analysis concentrated on the common DEGs,but not the first DEGs.

### Transcription factors and gene regulatory networks analysis

The expression of encoding genes has an apparent correlation with the cis and trans-regulatory elements^32^. Trans-regulatory elements (TRE) code the particular proteins called transcription factors which can highly influence the genes’ expression^33^. ChIP enrichment analysis (ChEA) database (https://maayanlab.cloud/chea3/) was used to identify the transcriptional factors that control the expression of common up- and downregulated genes of primary and metastatic tumor cells in comparison to normal cells. The information provided in the ChEA results from analysis of ChIP-based experimental methods such as ChIP-chip, ChIP-seq, ChIP-PET, and DamID which are used to profile the transcription factors that can bind to DNA and affect genes’ expression^34^. After selecting the most effective TFs overruling common DEGs regarding the adjusted *p*-value, the number of target genes and false discovery rate (FDR) were also calculated.

The common DEGs were also submitted to eXpression2Kinases (X2K) (https://amp.pharm.mssm.edu/X2K/) to find the gene regulatory network. X2K is an online bioinformatics resource that is generated to predict the relationship amongst upstream kinase pathways, the most influential TFs, and target genes^35^. It designs a diagram demonstrating the association of TFs, protein complexes, and protein kinases, which are responsible for the changes in the expression level of common up- and downregulated genes and transcriptome^35^. The inferred network of regulatory factors was downloaded and then visualized using Cytoscape software version 3.9.1 (https://cytoscape.org/).

### Detecting upregulated transcription factors

In order to discern common upregulated TFs, 573 common upregulated gene symbols as well as human TFs list including 1640 TFs’ names from The Human Transcription Factors database (http://humantfs.ccbr.utoronto.ca/)^36^ were uploaded to Venny (https://bioinfogp.cnb.csic.es/tools/venny/)^31^. The gene symbols common in both groups showed the common upregulated TFs. Since such TFs may play a crucial role in PPIs and other genes expression, they were separately detected for further prospective research. The same process was conducted to discover common downregulated TFs by uploading 690 common downregulated genes and human TFs to the Venny tool.

### Gene ontology, pathways, and functional analysis of DEGs

Enrichment analysis was conducted to probe a group of genes’ functions and their related pathways. This analysis also helped to understand the biological characteristics of the candidate genes, such as gene ontology (GO)^37^. Gene ontology analysis was applied to investigate the associated biological process (BP), cellular component (CC), and molecular function (MF) of common DEGs between primary and metastatic cancerous cells. This analysis was carried out using EnrichR (https://amp.pharm.mssm.edu/Enrichr/), a free online and web-based bioinformatics tool that comprehensively investigates the BP, CC, and MF related to submitted genes^38^.

The signaling pathways, in which upregulated and downregulated genes are contributed, are the other factors that can make a basis for understanding the tumorigenesis and metastasis process^39^. The Kyoto Encyclopedia of Genes and Genomes (KEGG, www.genome.jp/kegg/)^40–42^ website was used to analyze the particular pathways where the detected DEGs may play a crucial role. The KEGG database is an online resource for analyzing the pathways and functions of the inserted genes^40^. An adjusted *p* value < 0.05 was applied as a statistical index to detect the most meaningful GOs and pathways.

### Detection of DEG-targeting MicroRNAs and DEGs-related metabolites

Evidently, microRNAs (miRNA) are responsible for controlling the expression of various genes by targeting them in eukaryotic cells; therefore, they play one of the most crucial roles in transcriptome changes and can influence the level of gene expression in any disease^43^. miRTarbase (http://amp.pharm.mssm.edu) is a comprehensive free database which its 2020 update contained more than 13,404 validated miRNA-target interactions (MTIs)^44^. It provides diverse information on miRNAs which probably influence the submitted genes. It is widely used to detect transcriptome-affecting miRNAs, and its data are validated by experimental methods such as molecular assay, Northern blot, Microarray, and next-generation sequencing (NGS)^45^. The common DEGs between primary tumor and metastatic cancerous cells were submitted to this database and the outcomes were imported to Microsoft Excel (https://www.microsoft.com/en-us/download/details.aspx?id=56547) for further analysis. The top 10 miRNAs targeting up- and downregulated genes were selected based on their adjusted *p*-value.

It is testified that human biological metabolites can potentially affect the expression of different genes contributing to disorders like cancer^46^. This issue highlights the importance of surveying the most influential metabolites when investigating the transcriptome changes in diverse diseases^47^. The EnrichR database that is linked to Human Metabolome Database (HMDB)^48^ provides a broad domain of information on various metabolites affecting gene expression, along with their biological roles and disease associations. Therefore, this database was used to identify the top 10 important metabolites. A table was designed for the top 10 metabolites relevant to common up- and downregulated genes in primary and metastatic tumor cells by considering their *p-* value/adjusted *p*-value.

### Construction of PPI network, detection of hub genes, and modular analysis

The protein–protein interactions (PPI) among coding genes may have an essential role in cancer incidents and can also be utilized as targets for cancer treatment^49^. The common DEGs were inserted in the Search Tool for Retrieval of Interacting Genes database (STRING) (version 11.0; https://string-db.org), which gives remarkable information on both known and predicted PPI among coding DEGs^50^, to identify and anticipate functional interactions among expressed proteins by transcriptome (the medium confidence ≥ 0.400 was set as a cut-off to form the PPI network). The PPI networks for up- and downregulated genes were imported to Cytoscape software (version 3.9.1; www.cytoscape.org) for further visualization, modification, and analysis, such as module construction and hub genes detection.

Modules are described as a group of closely-associated genes that cooperate in arranging a particular function in PPI networks^51^. A Cytoscape plug-in named Molecular Complex Detection (MCODE) (version 2.0.0) was applied on PPI networks to illustrate the most critical gene modules by considering Degree Cutoff = 2, Node Score Cut-off = 0.2, K-Core = 2, Max. Depth = 100 and haircut cluster finding setting as visualization criteria^52^. The Cyohubba (version; 0.1), another Cytoscape software plug-in, was utilized for ranking and discerning the critical nodes (which are equivalent to hub genes) in the PPI networks^53^. The top 15 and 18 nodes (i.e., coding genes) of PPIs ranked by Maximal Clique Centrality (MCC) were ascertained as hub genes for common up- and downregulated genes between primary tumor and metastatic cancerous cells, respectively.

### Survival analysis, hub genes characterization, and study validation

In order to determine the prognostic importance of the detected hub genes and validate the study, the five top hub genes having the most meaningful relations with both primary and metastatic cancer cell lines (according to MCC analysis by Cytohubba) was submitted to the Kaplan–Meier plotter (https://kmplot.com/analysis/). Kaplan–Meier plotter is an online and free gadget primarily used for survival analysis in various cancers^54^. The GEPIA (http://gepia.cancer-pku.cn/), a bioinformatics web resource based on TCGA and GTEx data which provides comprehensive information on the level of different gene expression^55^, was also applied to investigate the expression of common hub genes in normal and tumor tissues and to authorize the results. The Human Protein Atlas database (https://www.proteinatlas.org/), a Swedish biological program which started in 2003 aiming at visualizing human histology by considering the expression of varied proteins in cells and tissues and integrating diverse omics data^56^, was used to illustrate the effect of the expression of proteins encoded by 5 top upregulated hub genes on normal and cancerous tissues.

### Cancer gene dependency and gene-disease association analysis

One crucial analysis that reveals a utile comprehensive outcome on cell lines’ survival at the gene level is cancer gene dependency. To evaluate the dependency rate of various breast cancer cell lines to identified upregulated hub genes on one hand, and validate the upshots on the other hand, the online platform DepMap, which can be accessed from https://depmap.org/portal/download was adopted^57^. To dive into details, the CRISPRGeneDependency.csv file was downloaded from DepMap Public 22Q4 Primary Files^58^. Then, the list of breast cancer cell lines was searched in the mentioned file, and the dependency score of 15 upregulated hub genes was extracted from it. In the next step, a text (.txt) file was composed of found data^59^. To draw the hub-gene dependency heat map, after using advanced options, the text file was uploaded to CIMMiner (one matrix CIM) (https://discover.nci.nih.gov/cimminer/oneMatrix.do)^60^. Eventually, the gene dependency heat map for 15 upregulated hub genes in primary and metastatic breast cancer cell lines was interpreted after downloading the final heat map^61^.

In the final step, to find out more about the potential role of detected upregulated hub genes, such genes’ characteristics were analyzed in a breast carcinoma file (UMLS CUI: C0678222; MeSH: D001943) downloaded from the DisGeNET database (https://www.disgenet.org/dbinfo)^62^.

### More investigations to find biological patterns in various transcriptomic data of breast cancer

In order to investigate the similar or different patterns that may be present among various types of biological samples, an identical analysis was conducted on a GSE65216 (GPL570) dataset. This dataset, publicly available since Jan 23, 2015^63^, contains expression profiling of diverse kinds of breast cancer from Institut Curie (Maire cohort). To find any similar or different patterns between this dataset (GSE65216) and the primary dataset of the study (GSE55715), the same analysis approach was carried out using the GEO2R tool by defining the triple-negative breast cancer (TNBC) samples as test group and healthy samples of this dataset as the control group. The results were then imported to Excel to find the upregulated genes in TNBC samples compared to healthy samples considering the same cut-offs (Log (adjusted *p*-value) < 0.05 and LogFc > 2). Afterward, the PPI network of the upregulated genes was elicited from the STRING database to find the hub genes for final comparison by exporting the data to the Cytoscape application and analyzing it by Cytohubba plug-in. The 20 top upregulated hub genes of GSE65216 were then aligned with 15 top upregulated hub genes of GSE55715 (as the primary dataset of this study) to discover any consistent results and similar patterns between the two datasets.

Furthermore, to make a comprehensive analysis of different types of breast cancer considering the varied PAM50 subtypes and to enhance the reliability of the found outcomes, one more analysis was performed on the GSE45827 dataset^64^. To dive into detail, PAM50 is one prominent categorization in which a 50-gene signature is utilized to classify the breast cancer in five different types in oncological studies^65,66^. Firstly, different breast cancer subtypes were divided into three separate test groups: Basal-like, Luminal B, and HER2-enriched. Shortly, Luminal B is a type of breast tumor with estrogen receptors, which can be progesterone negative or positive. The only difference between these two types is that Luminal B has HER2 receptors while Luminal B lacks them. HER2-enriched and basal-like tumors are both ER and PR negative, but HER2-enriched tumors are HER2 positive, while basal-like (also known as triple-negative breast cancer) is HER2 negative^67^. Subsequently, these three test groups were compared to the healthy samples as the control group. Then, the identical approach and cut-offs were followed to identify the upregulated genes, draw the PPI network, and indicate the top hub genes.

In both analyses, the volcano plot, as well as the UMAP diagram, were reviewed to identify the separation index of the test and control groups. Briefly, the more test and control samples are separated, the more reliable the analysis is due to the statistical principles.

### Breast cancer metastasis to various organs: Is there any rational relevance?

To investigate any rational relation between breast cancer metastasis to various organs, a comprehensive comparison ran among the common upregulated hub genes designated to have a critical role in primary and bone-metastatic breast cancer and the hub genes which were responsible for breast cancer metastasis to skin. The GSE56493 dataset appropriate samples were divided into two groups: 1. Skin-metastasis breast cancer as the test group, and 2. normal breast cells as the control group. Then, the same cut-offs (Log (adjusted *p*-value) < 0.05 and LogFc > 2), database (STRING), and plug-in (Cytohubba) were used to draw and detect the PPI network of upregulated hub genes, respectively. Afterward, the top 20 upregulated hub genes playing an essential role in breast cancer metastasis were compared to the top 15 common upregulated hub genes of GSE55715.

The tables, figures, and other data generated by applying mentioned methods are entirely presented in the “Results” section.

## Results

### Data analysis and common DEGs Identification

As mentioned before, this study was carried out to detect the most crucial genes that play a vital role in both primary tumor and bone-metastatic cancerous cells compared to normal mammary cells in breast cancer. The recognition of the most critical upregulated coding genes in both cancerous tissues can widely be used in Ducking and pharmacological studies to design an efficient therapeutic agents which are able to target both tumor cells simultaneously.

The gene expression (transcriptomes) changes in two test groups (primary (as Group 1) and bone-metastatic (as Group 2)) were compared with normal cells as a control group through the GEO2R tool in NCBI. The boxplot, volcano plot, and expression density plot (based on normal distribution) are available in Supplementary 1. The common DEGs of experimental groups, determined through cut-off condition adjusted *p*-value ≤ 0.05 and │(Log Fc)│ ≥ 2, were distinguished using the Venny online tool. Table 1 demonstrates the exact number of upregulated and downregulated genes in primary and metastatic tumor cells after applying the cut-off criteria. Venn diagram of the common DEGs among groups 1 and 2 can be seen in Supplementary 2.

**Table 1:** Number of DEGs in primary/metastatic tumor cells in comparison to normal cells.

Group number	Experimental versus control groups	DEG type	Number of genes
Group 1	Primary tumor cell versus Normal cell	Upregulated	889
		Downregulated	946
Group 2	Metastatic tumor cell versus Normal cell	Upregulated	1131
		Downregulated	1180
The number of common upregulated genes between Group 1 and 2			573
The number of common downregulated genes between Group 1 and 2			690

### 10 TFs associated with common up- and down regulated genes and gene regulatory networks

Since TFs can widely impact gene expression, the common up- and downregulated genes between groups 1 and 2 were separately submitted to ChEA 2016 database to find the potential TFs. All TFs that had adjusted *p-*value ≤ 0.05 were considered effective TFs having meaningful relationships with the submitted genes. This indicates that 59 human TFs are capable of targeting both primary and metastatic up regulated genes. In contrast, there were only 15 potential human TFs that could target common down regulated genes between the defined groups. ETS1 20019798, GABP 19822575, and AR 21909140 were the top 3 most influential TFs for common upregulated genes, while VDR 23849224, SOX2 20726797, and CHD1 26751641 were detected as the top 3 important TFs for common downregulated genes influencing at least 106 genes. The top 10 transcription factors associated with common DEGs between groups 1 and 2 are displayed in Table 2.

**Table 2:** Top ten TFs binding to common up- and downregulated genes between primary and metastatic tumor cell.

Type of differentiation	TFs terms	TF description	Number of targets	Adjusted *p* value	FDR
Upregulated genes	ETS1 20019798	Erythroblast transformation specific proto-oncogene1 transcription factor 20019798	86	1.03E−06	1.13E−07
	GABP 19822575	GA-binding protein transcription factor 19822575	122	2.34E−06	1.86E−07
	AR 21909140	Androgen receptor transcription factor 21909140	28	2.34E−06	1.35E−07
	RUNX1 21571218	Runt-related transcription factor-1 21571218	199	1.08E−05	6.38E−07
	E2F7 22180533	E2F transcription factor-7 22180533	10	2.21E−05	1.11E−06
	FOXM1 25889361	Forkhead box protein M1 transcription factor 25889361	54	2.77E−05	1.23E−06
	FLI1 21571218	Friend leukemia integration 1 Transcription factor 21571218	220	3.33E−05	1.34E−06
	SPI1 23547873	Spi-1 proto-oncogene transcription factor 23547873	134	6.32E−05	2.48E−06
	XRN2 22483619	5′–3′ exoribonuclease 2 transcription factor-1 22483619	75	8.78E−05	3.47E−06
	FOXM1 23109430	Forkhead box protein M1 transcription factor 23109430	22	2.40E−04	9.80E−06
Downregulated genes	VDR 23849224	Vitamin D Receptor Transcription Factor 23849224	130	6.53E−07	1.02E−08
	SOX2 20726797	Sex-determining region Y-boX-2 transcription factor 20726797	140	4.54E−06	7.09E−08
	CHD1 26751641	Chromodomain helicase DNA binding protein-1 transcription factor 26751641	106	9.77E−04	1.53E−05
	AR 21915096	Androgen receptor transcription factor 21915096	103	0.002744873	5.36E−05
	AR 21909140	Androgen receptor transcription factor 21909140	25	0.004513302	8.46E−05
	ZNF217 24962896	Zinc-finger protein-217 transcription factor 24962896	81	0.005275506	1.01E−04
	HNF4A 19822575	Hepatocyte nuclear factor 4 alpha transcription factor 19822575	256	0.005275506	1.11E−04
	RELA 24523406	v-rel avian reticuloendotheliosis viral oncogene homolog A transcription factor 24523406	64	0.010726215	2.93E−04
	SMAD4 19686287	Mothers against decapentaplegic homolog 4 transcription factor 19686287	28	0.016549252	4.60E−04
	ELK1 19687146	ETS transcription factor ELK-1 19687146	51	0.019229791	5.43E−04

**Figure 2:**
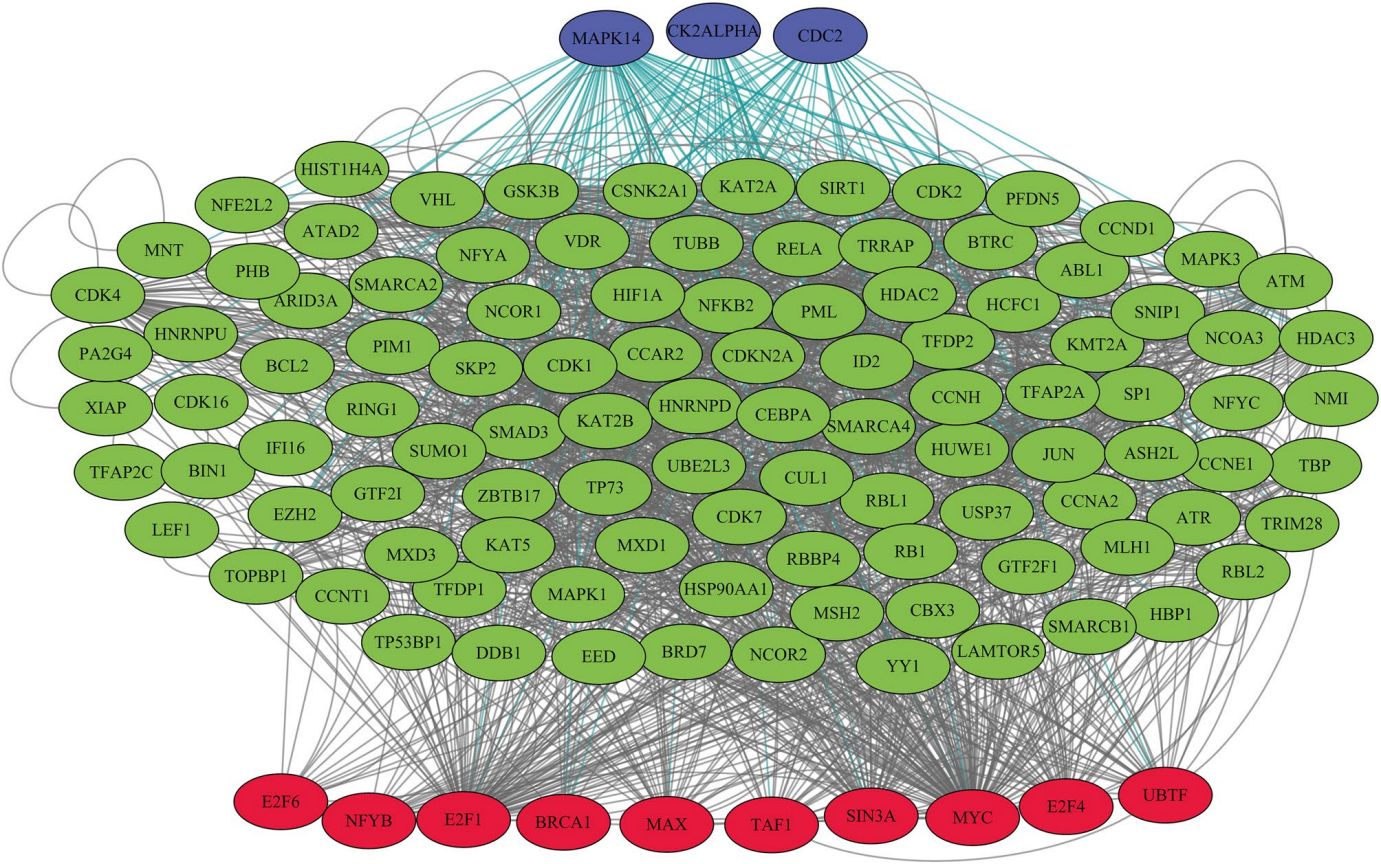
Gene regulatory network common upregulated genes in primary tumor and metastatic cancerous cells involving TFs and kinases.

X2K web-based bioinformatics resource was used to design the gene regulatory networks and identify the role of the detected TFs by the ChEA 2016 database more accurately. The results of investigating 573 common upregulated genes revealed that the enzymes encoded by Mitogen-Activated Protein Kinase-14 (MAPK14), cell division control-2 (CDC2), and casein kinase two alpha-1 (CK2ALPHA) were the most substantial kinases in the regulatory network of the common upregulated genes. These findings give credence to the results elicited from ChEA 2016 by approving the role of MYC and E2F1 TF families. The gene regulatory network of common upregulated DEGs of groups 1 and 2 is illustrated in Fig. 2.

**Figure 3:**
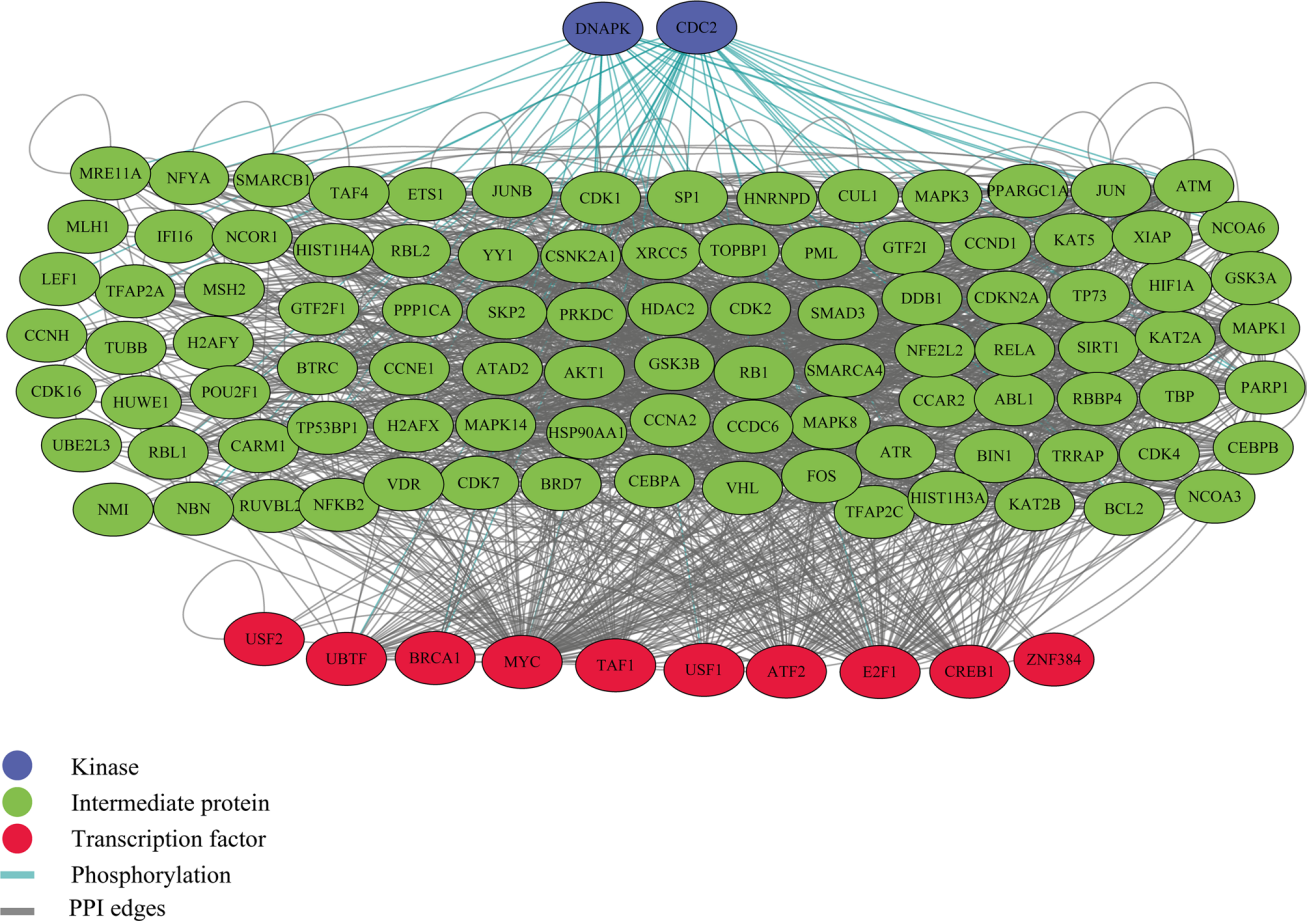
Regulatory network of common downregulated genes in both tumor groups containing TFs and kinases.

The same method was developed for investigating the gene regulatory networks of common downregulated genes, eventually elaborating the importance of the DNA-dependent protein kinase (DNA-PK) and CDC2 kinases in the network. The roles of TFs were also explicitly shown in the X2K database diagram. The modified gene regulatory network for common downregulated genes is depicted in Fig. 3.

### Upregulated transcription factors

**Figure 4:**
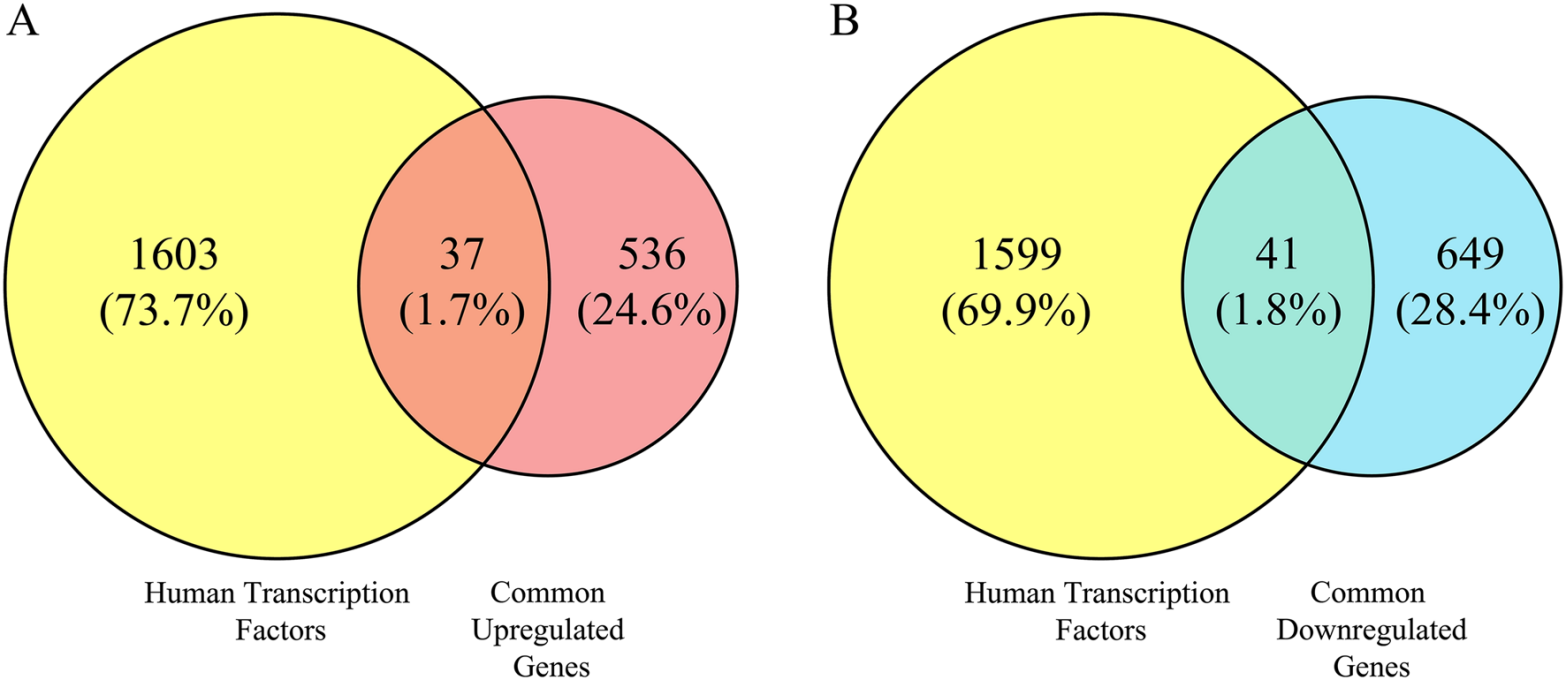
Venn diagram of applied method to detect common (**A**) up- and (**B**) downregulated TFs. Exact gene symbols of common differentially expressed transcription factors are listed in Supplementary 3.

As previous research has indicated, transcription factors–as a group of proteins highly involved in transcribing DNA and RNA synthesis—can broadly influence the expression level of their downstream genes by initiating or regulating such genes’ transcription^68^. As a result, detecting the upregulated TFs amongst all common upregulated genes can be used to illuminate unknown networks and signaling pathways. As previously mentioned, highlighting the transcription factors among DEGs and other analyses are crucial since such TFs have the regulatory effect (like co-expression, suppression or activation) on the other genes^36^. Moreover, the mode of regulation of such TFs also reveal the PPI network more accurately^69,70^. Figure 4 demonstrates the number and percentage of common upregulated (A) and downregulated (B) transcription factors.

### The results of gene ontology and KEGG pathway analysis

**Figure 5:**
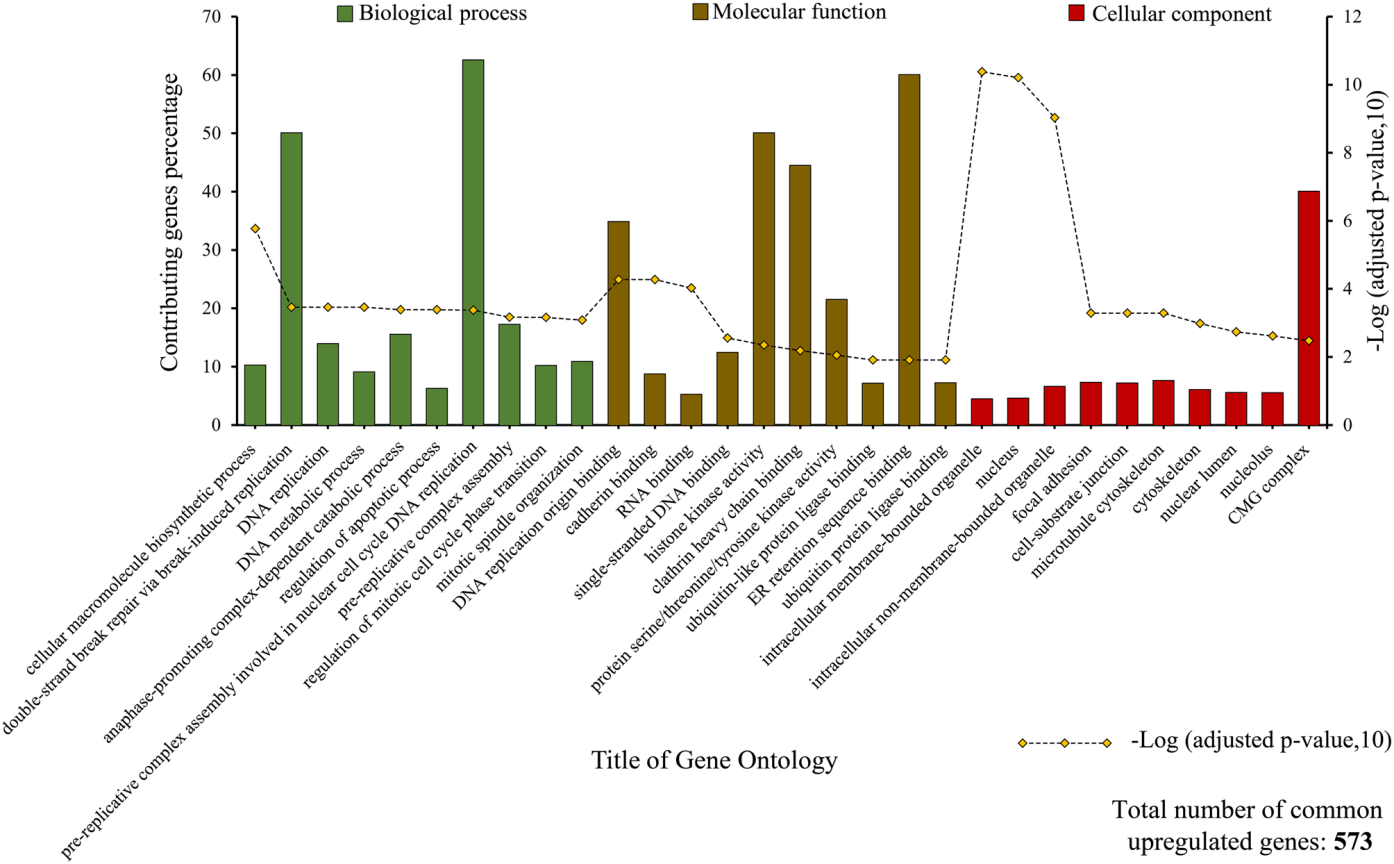
GO enrichment analysis of common upregulated genes between group 1 (primary breast cancer vs. normal) and group 2 (bone-metastatic breast cancer).

After analyzing the GSE55715 dataset and separating the common DEGs between groups 1 and 2, the GO of 573 common upregulated genes and 690 common downregulated genes were investigated by EnrichR GO 2021 online resource. It was found that common upregulated genes primarily contributed to DNA-related biological processes (BP) such as the double-strand break repair and DNA replication. GO molecular function analysis also revealed that binding to DNA replication origin, cadherin, RNA, and single-strand DNA was the most associated molecular functions to which a considerable number of these 573 genes were contributed. In addition, common upregulated genes were enriched in the different cellular components (CC). Nucleus, focal adhesion, and cytoskeleton are some instances of these CCs. The top 10 most critical GO in which common upregulated genes were contributed, along with the proportional percentage of involved genes, are demonstrated in Fig. 5.

**Figure 6:**
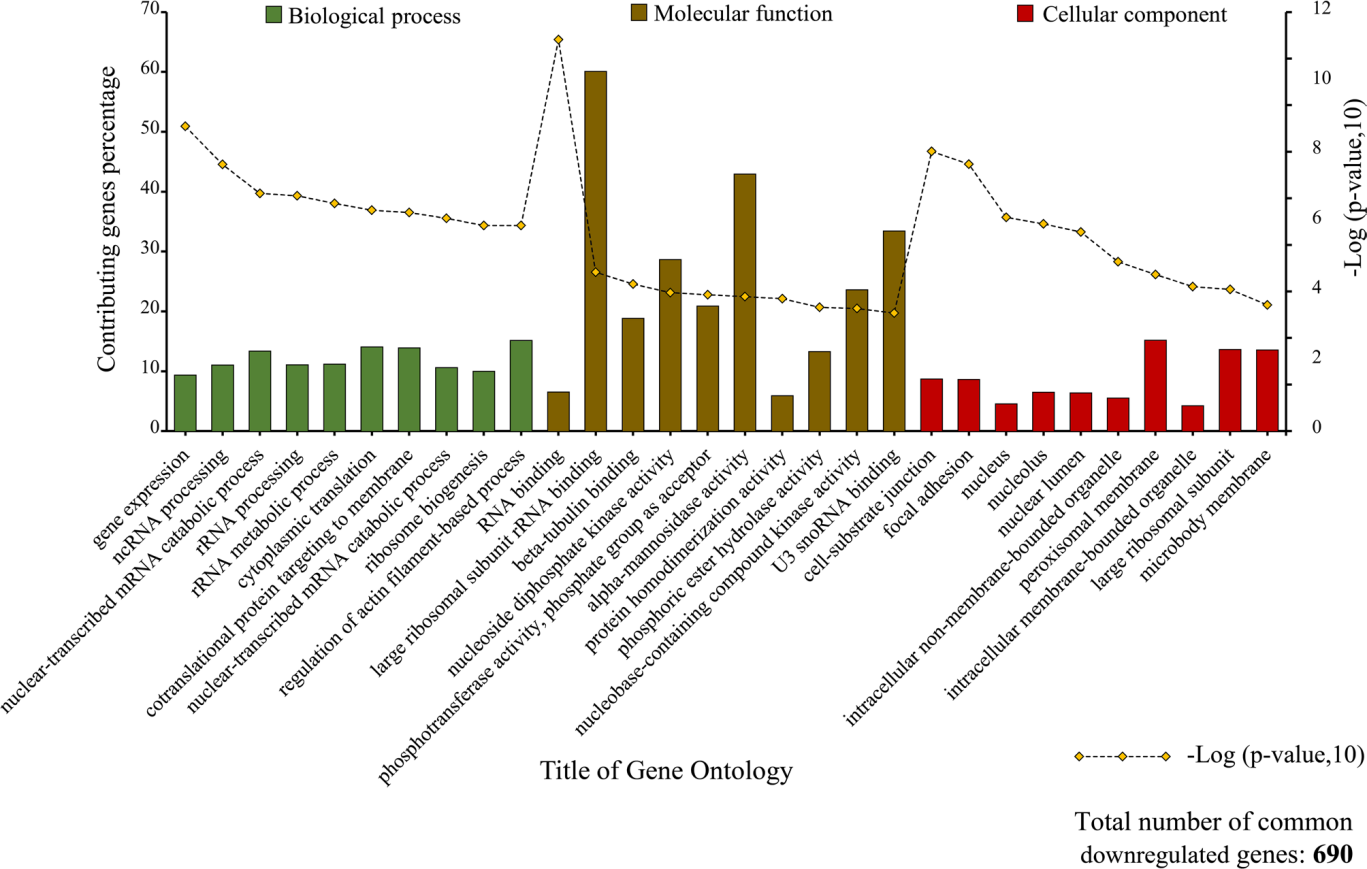
GO enrichment analysis of common downregulated genes between defined groups.

The GO investigation of common downregulated genes by the EnrichR 2021 GO database unveiled that gene expression, ncRNA processing, and nuclear-transcribed mRNA catabolic process were the most associated BP with some of the 690 down regulated genes. It was also displayed that the most influential MF and CC in which common downregulated genes were involved. The GO diagram of common downregulated genes can be observed in Fig. 6.

**Figure 7:**
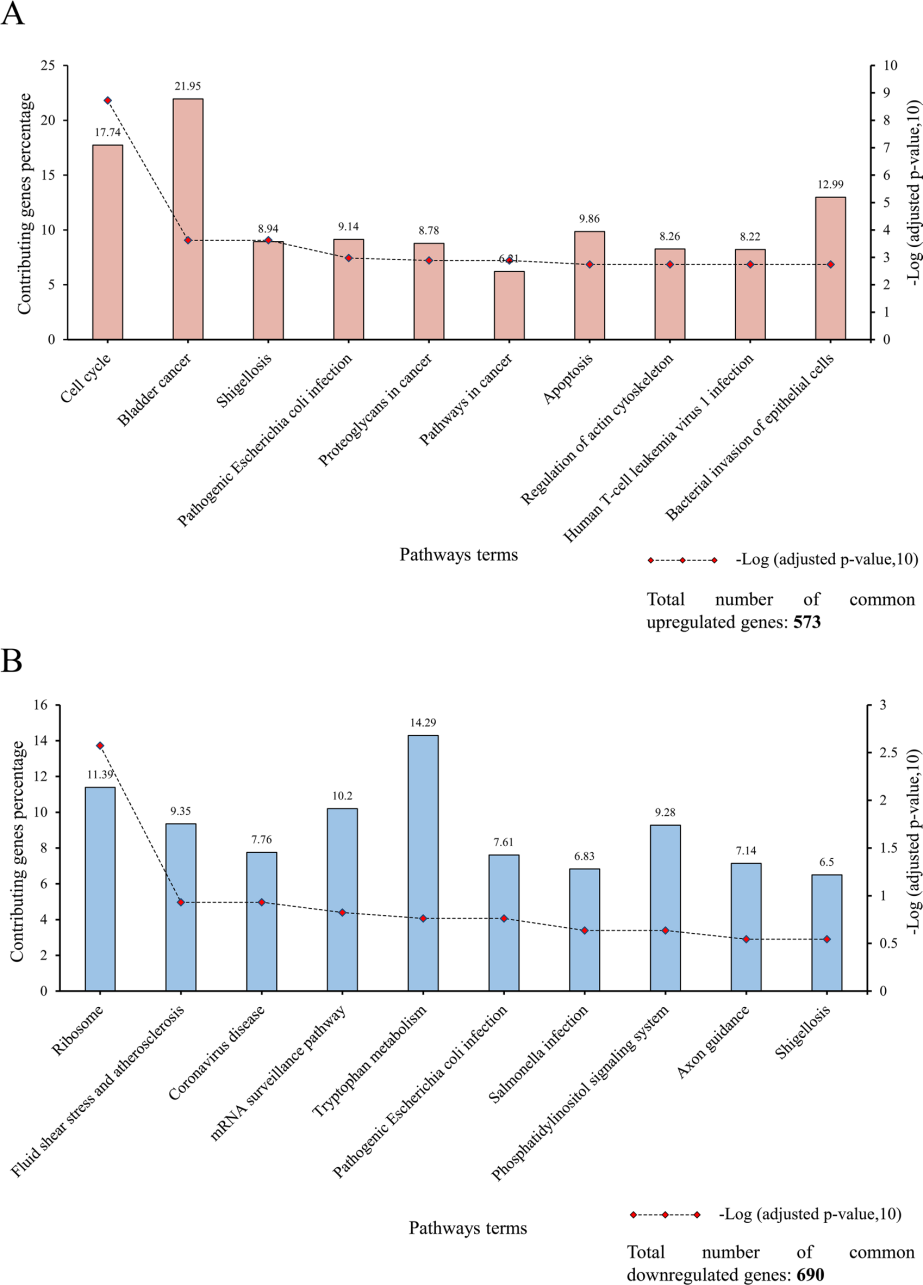
Top 10 KEGG pathways in which common upregulated genes (**A**) and common downregulated genes (**B**) play an essential role.

In terms of function and pathways, the analysis of common DEGs by the KEGG database was used, and the most prominent pathways were determined by considering adjusted *p*-value ≤ 0.05. The findings indicate that common upregulated genes were primarily involved in the cell cycle and bladder cancer-related pathways (Fig. 7A). In contrast, common downregulated genes were remarkably enriched in ribosome or infection-related pathways (Fig. 7B).

### Finding miRNA-targeting genes and common DEGs-related metabolites

Due to the importance of miRNA-targeting genes and metabolites at the level of various genes, miRTar base 2017 and EnrichR HMDB online databases were utilized to identify the most effective miRNAs and metabolites, respectively. The complete list of miRNA-targeting genes was downloaded, and the influential ones were detected by considering the adjusted *p*-value ≤ 0.05. It is shown that hsa-miR-615-3, hsa-miR-124-3p, and hsa-miR-92a-3p have the most meaningful relationships with common upregulated genes by targeting 67, 87, and 83 genes, respectively. In Table 3, the top 10 most critical miRNA-targeting genes, along with the number of their target genes (among the submitted genes) and overlap percentage, are exhibited.

**Table 3:** Top ten miRNAs targeting the common DEGs between primary and metastatic tumor cells.

Type of DEGs	miRNA	Number of targets	Overlap percent of this miRNA’s targets with submitted genes (%)	Adjusted *p* value
Upregulated genes	hsa-miR-615-3p	67	7.52	1.33E−09
	hsa-miR-124-3p	87	6.02	3.75E−08
	hsa-miR-92a-3p	83	5.91	1.90E−07
	hsa-miR-34a-5p	52	7.07	1.40E−06
	hsa-miR-193b-3p	57	6.69	1.40E−06
	hsa-miR-16-5p	82	5.27	2.33E−05
	hsa-miR-222-3p	32	8.12	5.39E−05
	hsa-miR-1-3p	55	5.98	7.50E−05
	hsa-miR-186-5p	47	6.27	1.37E−04
	hsa-miR-877-3p	40	6.60	2.62E−04
Downregulated genes	hsa-miR-215-5p	55	7.28	4.28E−04
	hsa-miR-16-5p	91	5.85	4.30E−04
	hsa-miR-192-5p	65	6.55	4.30E−04
	mmu-miR-181a-5p	38	8.30	4.30E−04
	hsa-miR-128-3p	40	7.59	0.001643017
	hsa-miR-30a-5p	49	6.68	0.003819381
	mmu-miR-340-5p	41	7.12	0.004220497
	hsa-miR-183-5p	29	8.26	0.00435358
	hsa-miR-32-5p	40	7.08	0.00435358
	hsa-miR-92b-3p	47	6.63	0.00435358

After the analysis of common DEGs amongst the sorted groups by EnrichR HMDB, the most crucial metabolites influencing the expression of genes were identified according to their *p*-value (*p* value ≤ 0.05). Overall, 1080 metabolites were detected to have a reliable association with various common upregulated genes, whereas only three metabolites (i.e., 1H-Indole-3-acetaldehyde, 5-hydroxy-Sulfate, and TYD) unveiled a cogent relationship with common downregulated genes. Guanosine triphosphate, uridine Diphosphate-N-acetyl glucosamine, phosphate, and other phospholipids moiety were detected as the top 10 metabolites that affect gene expression. Table 4 presents the significant metabolites having strongly meaningful association with common DEGs.

**Table 4:** Top 10 metabolites associated with common DEGs between primary tumor and metastatic tumor cells.

Type of DEGs	Metabolite name	*P* value	Targeted genes
Up regulated genes	Guanosine triphosphate	0.0017913	*CYFIP1;RTKN;ROCK2;MAPKAP1;RAP1GDS1;NOLC1;MTIF2;FNBP1L;ABR;RBM4;NRAS;TUBA1A;TUBB3;GMPPA;RCC1;ADSS;RANBP3;EXOC7;TUBB;EIF5;EEF1D;GNAS;KRAS;KIF20A;BLZF1*
	Uridine diphosphate-N-acetylglucosamine	0.0052261	*DPAGT1;EXT2;MGAT4B;MGAT2*
	Phosphate	0.0277204	*PFKFB3;PNPT1;DUSP19;PTEN;ATP2B4;ATP5F1;CDC25A;ACACA;PPM1G;PPM1B;KATNAL1;KATNA1;GMPPA;UPP1;ADSS;ATP9A*
	PE(O-18:1(1Z)/20:4(5Z,8Z,11Z,14Z))^a^	0.0316249	*PLSCR3;PEMT;CEPT1;ATP9A*
	PE(O-16:1(1Z)/22:6(4Z,7Z,10Z,13Z,16Z,19Z))^b^	0.0316249	*PLSCR3;PEMT;CEPT1;ATP9A*
	PE(14:0/14:0)^c^	0.0316249	*PLSCR3;PEMT;CEPT1;ATP9A*
	PE(14:0/14:1(9Z))^d^	0.0316249	*PLSCR3;PEMT;CEPT1;ATP9A*
	PE(14:0/15:0)^e^	0.0316249	*PLSCR3;PEMT;CEPT1;ATP9A*
	PE(14:0/16:0)^f^	0.0316249	*PLSCR3;PEMT;CEPT1;ATP9A*
	PE(14:0/16:1(9Z))^g^	0.0316249	*PLSCR3;PEMT;CEPT1;ATP9A*
Down regulated genes	1H-Indole-3-acetaldehyde, 5-hydroxy	0.0071242	*ALDH3A2;MAOB;MAOA*
	Sulfate	0.0074888	*SLC26A2;MGST1;GSTT1;ARSD;PAPSS1*
	TYD	0.0111978	*DTYMK;NME2;NME1*

^a^Glycerophospholipid (modified in phosphorylethanolamine moiety).

^b^1-Palmitoleoyl-2-docosahexaenoyl-sn-glycero-3-phosphoethanolamine.

^c^Phosphatidylethanolamine (modifications in phosphorylethanolamine moiety).

^d^1-(9Z-tetradecenoyl)-2-tetradecanoyl-glycero-3-phosphoethanolamine.

^e^1-Myristoyl-2-pentadecanoyl-sn-glycero-3-phosphoethanolamine.

^f^1-Myristoyl-2-palmitoyl-sn-glycero-3-phosphoethanolamine.

^g^1,2-Dimyristoleoyl-rac-glycero-3-phosphoethanolamine.

### Protein–protein interactions, hub genes networks, and modules

**Figure 8:**
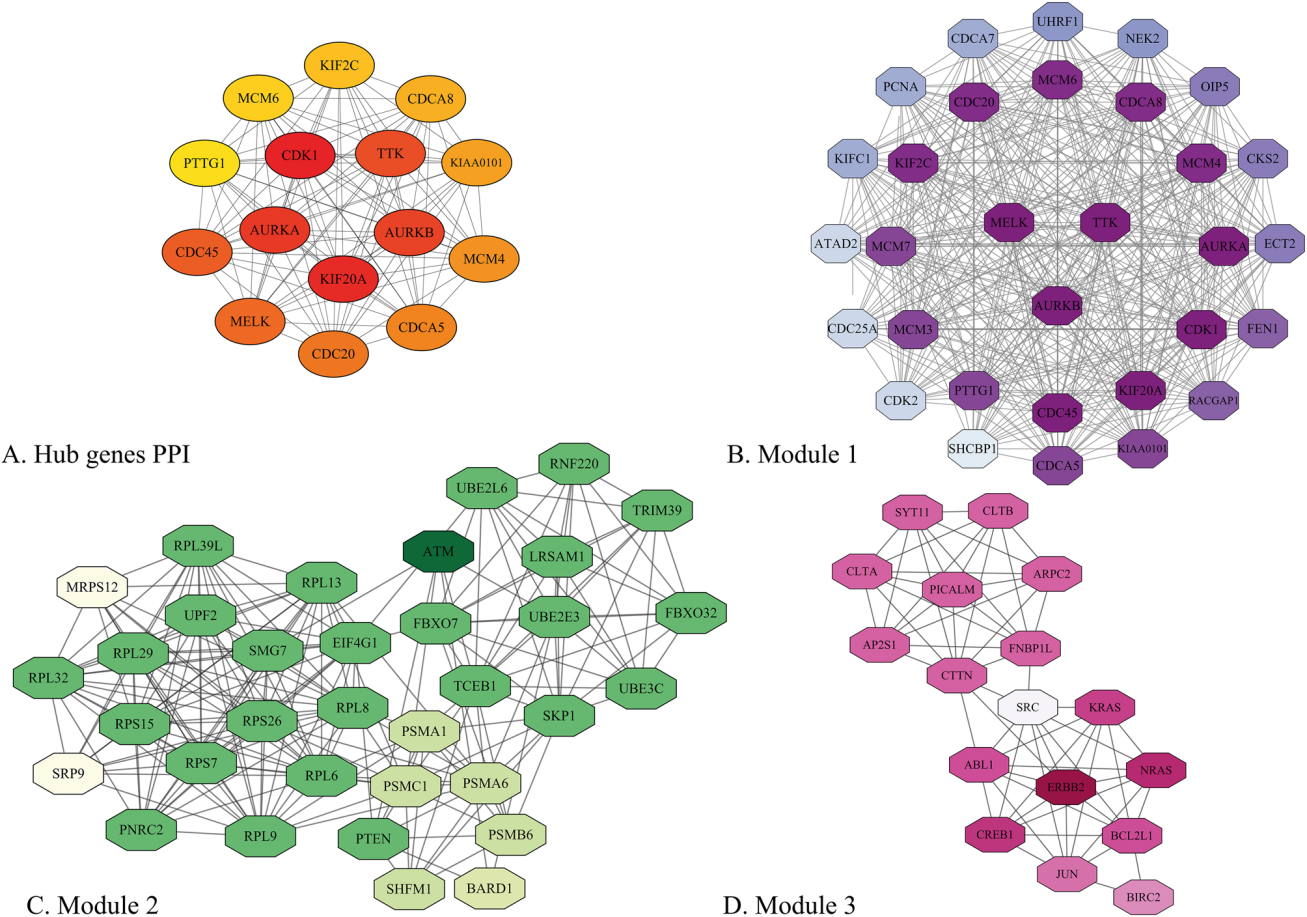
Common upregulated hub genes network of breast cancer-related genes (**A**), graphic illustration of Module 1 (**B**), 2 (**C**), and 3 (**D**). The stronger the color is, the more important that gene is.

The gene symbol of common DEGs was uploaded to STRING online bioinformatics resource to identify the PPI networks. As a result, STRING detected 560 out of 573 common upregulated genes as protein-coding genes, and their PPI enrichment *p*-value computed to be < 1.0e−16. The PPI network was then imported to Cytoscape for final visualization and analysis (which showed that the PPI network has 493 nodes and 3052 edges). The Cytoscape analysis revealed that 67 nodes (coding genes) were isolated and did not have any edges (interactions) with others, whereas 15 nodes had more than 46 degrees and were surprisingly dense. The PPI network of common upregulated genes is presented in Supplementary 4. The Cytohubba analysis demonstrated that most of these highly-connected genes, such as *AURKA*, *AURKB*, *MELK*, *TTK*, *KIF20A*, *CDK1*, *KIF2C*, *CDCA8*, *KIAA0101* (also known as *PCLAF*), *MCM4*, *CDCA5*, *CDC20*, *CDC45*, *PTTG1*, and *MCM6* are from hub genes. Overall, these 15 genes were detected as the hub genes by Cytohubba MCC analysis (approximately 2.6% of all common upregulated genes). Furthermore, the MCODE plug-in was also applied to identify the most significant modules, which reveals the three important modules, including Module 1 (31 nodes and 418 edges), Module 2 (34 nodes and 208 edges), and Module 3 (17 nodes and 62 edges). These three modules, as well as 15 hub genes, are displayed in Fig. 8.

**Figure 9:**
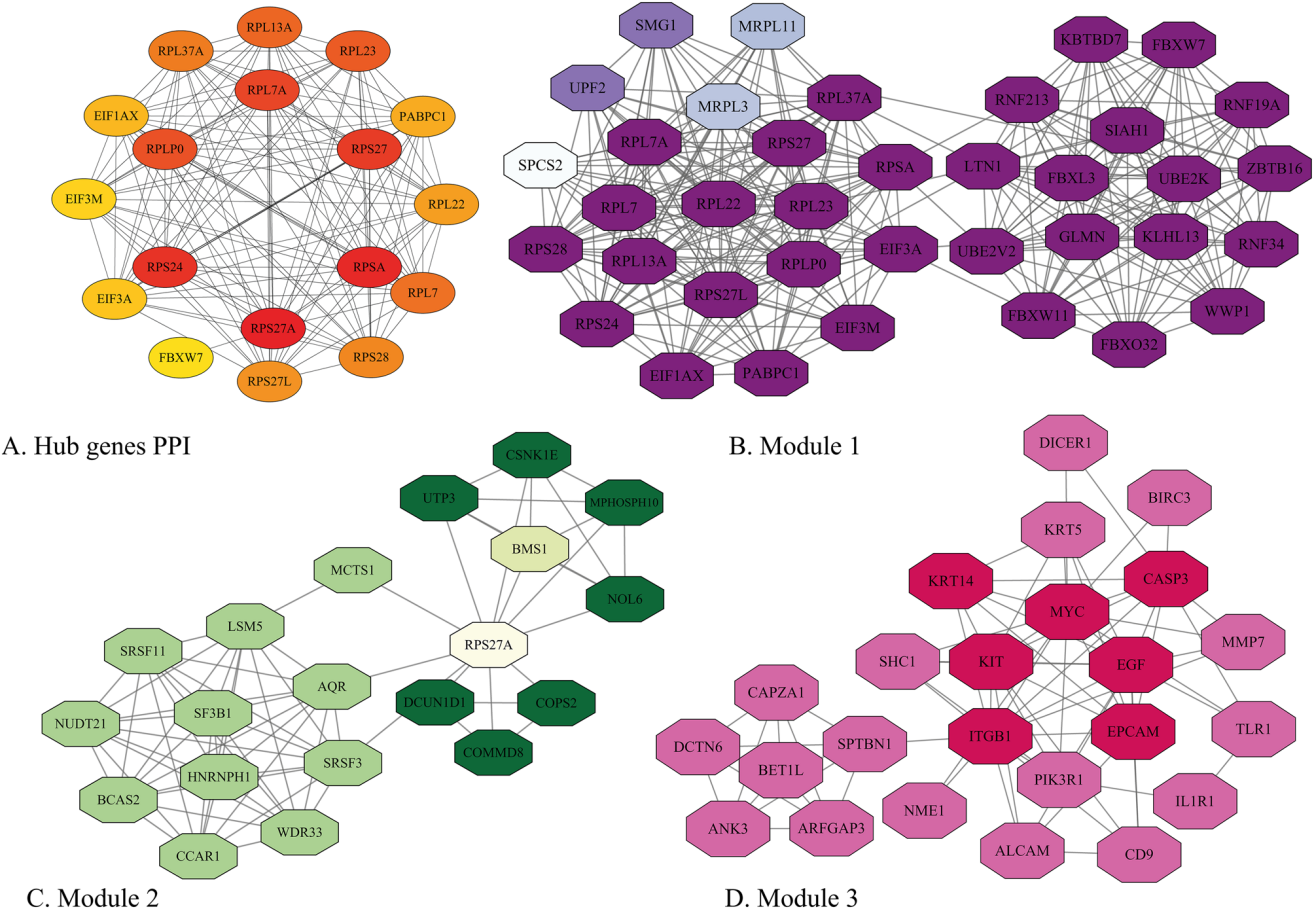
Common downregulated hub genes network (**A**), graphic presentation of Module 1 (**B**), 2 (**C**), and 3 (**D**). The bolder the color is, the more critical that gene is.

The all of 690 common downregulated genes among the primary and bone-metastatic tumors were submitted to STRING to find their PPI network. STRING detected 679 out of 690 genes and presented their PPI by reporting their PPI enrichment *p*-value to be 1.23e−12. The imported PPI network to Cytoscape had 2309 edges and 606 nodes, which indicated there are 73 isolated nodes in PPI, while 17 nodes had 31–86°. The entire PPI network of common downregulated genes is depicted in Supplementary 5. Alike common upregulated genes, 18 genes (about 2.6% of all genes) were identified as hub genes by Cytohubba, and their network was designed. In addition, we could find the most relevant gene groups by analyzing the whole PPI network by MCODE, which provided three critical modules. The common downregulated hub genes and Module 1 (37 nodes and 312 edges), Module 2 (20 nodes and 70 edges), and Module 3 (24 nodes and 75 edges) are shown in Fig. 9.

### Results of the survival analysis and study validation

**Figure 10:**
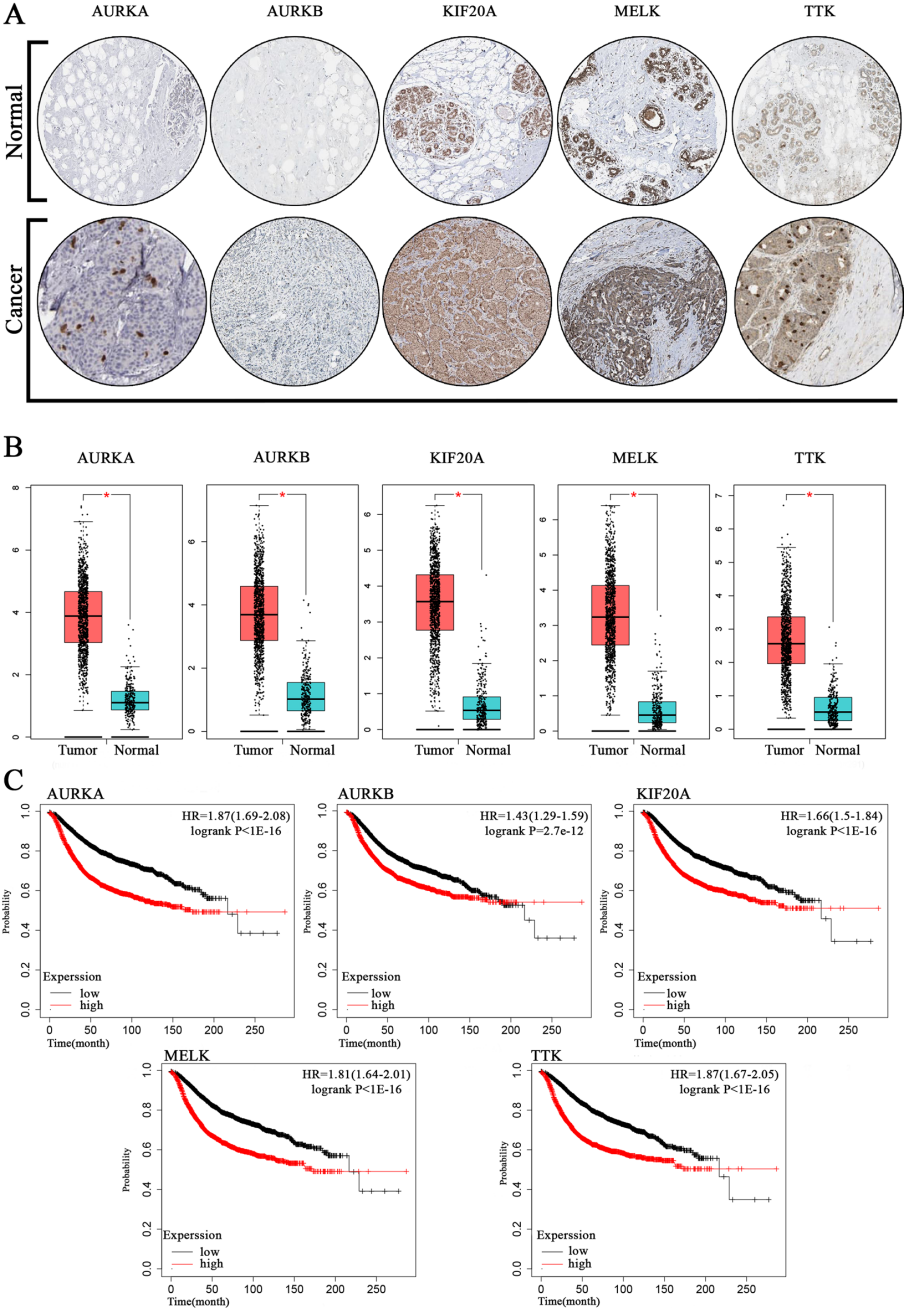
The results of validating study: Immunohistochemistry of the top five densest hub genes in breast carcinoma and normal tissue based on (**A**), the expression level of these five genes in healthy people and patients elicited from GEPIA (**B**), and survival analysis diagrams for commonly up regulated hub genes designed by Kaplan–Meier (**C**).

The validation of the results is the most quintessential part of the study, which can endorse the reliability of the data and assure researchers to conduct lab practices based on the results of this study. As explained in the methods section, the top five most pivotal upregulated hub genes were submitted into three various databases, including GEPIA, human protein atlas, and Kaplan–Meier plotter, to investigate the proportional amount of these common hub genes upregulation, their effect on tissue construction and survival rate, respectively. *AURKA*, *AURKB*, *KIF20A*, *MELK*, and *TTK* were identified as the most crucial hub genes based on their score after PPI analysis by the MCC method of Cytohubba plug-in. The human atlas protein analysis showed the appearance of abnormalities in tissue after the upregulation of these genes in tumor tissues. Moreover, GEPIA analysis indicates that these five genes were strongly upregulated, and their expression in patients was at least three times more than that of the healthy people. The Kaplan–Meier analysis also validated the reached results and demonstrated that these 5 top common hub genes were identified correctly. It also revealed that the survival rate of patients detected to have a higher level of these genes’ expression was dramatically lower than the control group [Hazard ratio (HR) index and Log (rank *p*-value) were entirely meaningful]. Overall, evaluating 5 candidate hub genes by these three databases thoroughly validated achieved results. Figure 10 illustrates the results of the validation process by human atlas protein (A), GEPIA (B), and Kaplan–Meier plotter (C).

### Cancer-gene dependency analysis of hub genes

**Figure 11:**
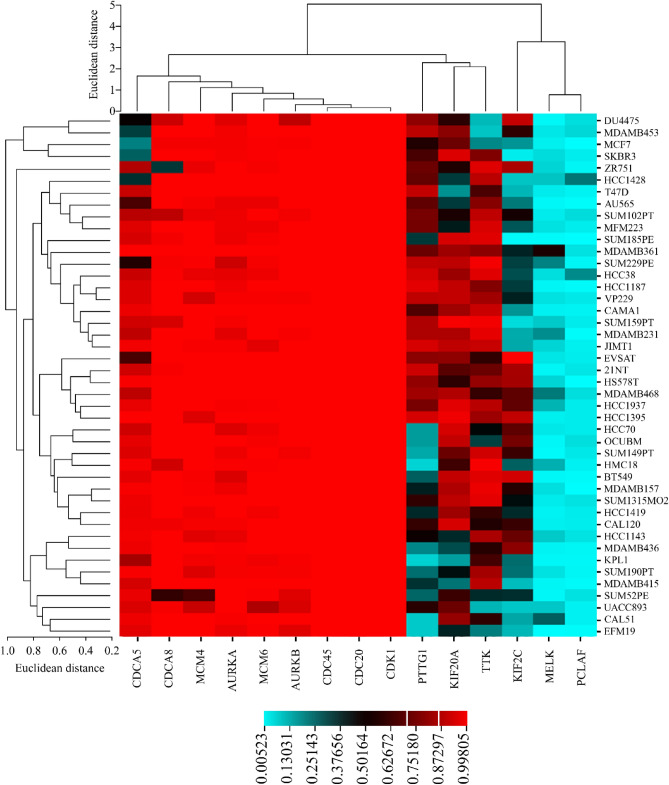
Heat map of gene dependency for primary and metastatic breast cancer and the 15 upregulated hub genes: while most of the hub genes are highly demanded for cell lines’ survival, some of them, including *MELK* and *KIAA0101* (*PCLAF*), are reportedly less vital for almost all of the cell lines (Right column: cell lines’ names, Row: hub genes’ symbols). The warmer (redder) a color is, the more vital that gene is for the corresponding cell line.

A cancer genetics dependency for a particular gene denotes how essential that gene is for the survival/proliferation of that cell line^58^. This is primarily computed by knocking out that gene in a cell line or inhibiting the protein encoded by a gene to measure its effect on blocking that cell line’s survival or inducing its death^71^. As expounded, the dependency score of 15 upregulated hub genes for available breast carcinoma cell lines amongst all cancer cell lines (CCL) was extracted from CRISPRGeneDependency.csv downloaded from the DepMap portal (https://depmap.org/portal/download/all/). Since cancer cell lines are broadly used as in vitro models for cancer-biology-related topics such as genes’ expressions, drug efficacy etc.^72^, measuring the genes expression and dependency in such cell lines can be used to validate or reject the findings. In the gene heat map, the redder a common point between a particular gene and a cell line, the more that cell line’s survival depends on that gene. The text of elicited data, including 15 upregulated hub genes, breast cancer primary and metastatic cell lines, and their dependency score (Supplementary 6), was used to draw the heat map in Fig. 11. As can be seen in Fig. 11, the dependency score of many breast carcinoma cell lines for most of the hub genes is high, indicating that those genes are vastly required for cells’ proliferation/survival. This heat map not only validates the integrity of found hub genes but also highlights the significance of discovered PPI networks, gene expression, and cellular signaling.

### Gene-disease relations for hub genes

In the final analysis of study validation, the DisGeNet web-based tool (https://www.disgenet.org/search) got used for the assessment of the gene-disease association for the top 15 upregulated hub genes to investigate the Disease-Specificity and Disease Pleiotropy indexes (DSI and DPI, respectively) as well as former articles which have reported that particular gene and breast carcinoma^62^. The DSI is a value between 0 and 1, indicating the number of diseases the gene is associated with in an inverse proportion, while DPI shows the variety of disease types in which that gene plays a role. In other words, the more DPI is the more types of disorders that gene is associated with, whereas the less DSI is, the more number of diseases are known to be related to that gene^73^. Table 5 provides a broad range of such information for common upregulated hub genes.

**Table 5:** Gene-disease association (GDA) for breast carcinoma and 15 common upregulated hub genes.

Gene symbol	Gene full name	Protein class	DSI	DPI	Number of PubMed articles supporting GDA	First and last year of GDA report
*AURKA*	Aurora kinase A	Kinase	0.475	0.731	51	1997–2019
*AURKB*	Aurora kinase B	Kinase	0.51	0.769	6	2007–2019
*CDC20*	Cell division cycle 20	Enzyme modulator	0.587	0.577	7	2014–2019
*CDC45*	Cell division cycle 45	Enzyme modulator	–	–	–	–
*CDCA5*	Cell division cycle associated 5	–	0.563	0.808	1	2018–2018
*CDCA8*	Cell division cycle associated 8	–	0.659	0.5	3	2018–2019
*CDK1*	Cyclin dependent kinase 1	Kinase	0.482	0.808	17	1996–2019
*KIF20A*	Kinesin family member 20A	Cellular structure	0.615	0.615	1	2016–2016
*KIF2C*	Kinesin family member 2C	Cellular structure	0.666	0.308	4	2007–2019
*MCM4*	Minichromosome maintenance complex component 4	Enzyme	–	–	–	–
*MCM6*	Minichromosome maintenance complex component 6	Enzyme	0.666	0.615	2	2019–2019
*MELK*	Maternal embryonic leucine zipper kinase	Kinase	0.566	0.577	9	2007–2019
*PCLAF (KIAA0101)*	PCNA clamp-associated factor	Chromatin binding activator	0.538	0.769	5	2011–2018
*PTTG1*	PTTG1 regulator of sister chromatid separation	–	0.526	0.654	13	2005–2019
*TTK*	Threonine and tyrosine protein kinase	Kinase	0.555	0.731	9	2006–2019

### Similar biological patterns in diverse transcriptomic data of breast cancer

As aforesaid, to make the study more comprehensive and endorse its reliability, two more analyses were done on other datasets, including GSE65216 and GSE45827. The results from the first analysis indicated that five upregulated hub genes were shared between GSE65216 and GSE55715, of which 4 of them were previously introduced as the most critical genes having a crucial role in both primary and bone-metastatic breast cancer. This significant overlap emphasizes the primacy of drug discovery or designing for proposed genes in this study. The full results of this analysis are attached in Table 6.

**Table 6:** The hub upregulated genes in GSE65216 and GSE55270.

The 20 top upregulated hub genes in GSE65216 (in alphabetic order)	The 15 common upregulated hub genes in GSE55270	The shared hub genes between GSE65216 and GSE55279*
*ASPM, AURKB, BUB1B, CCNA2, CCNB1, CCNB2, CDK1, CENPF, DLGAP5, HMMR, KIF11, KIF20A, MELK, NCAPG, NUSAP1 RRM2, TOP2A, TPX2, TTK ZWINT*	*CDCA5, CDCA8, NCM4, MCM6, AURKA, AURKB TTK, MELK, CDC45, CDC20, CDK1, PTTG1, KIF20A, KIF2C PCLAF*	***AURKB*** *CDK1* ***KIF20A*** ***MELK*** ***TTK***

*The most critical genes are highlighted in bold according to the previous results.

**Figure 12:**
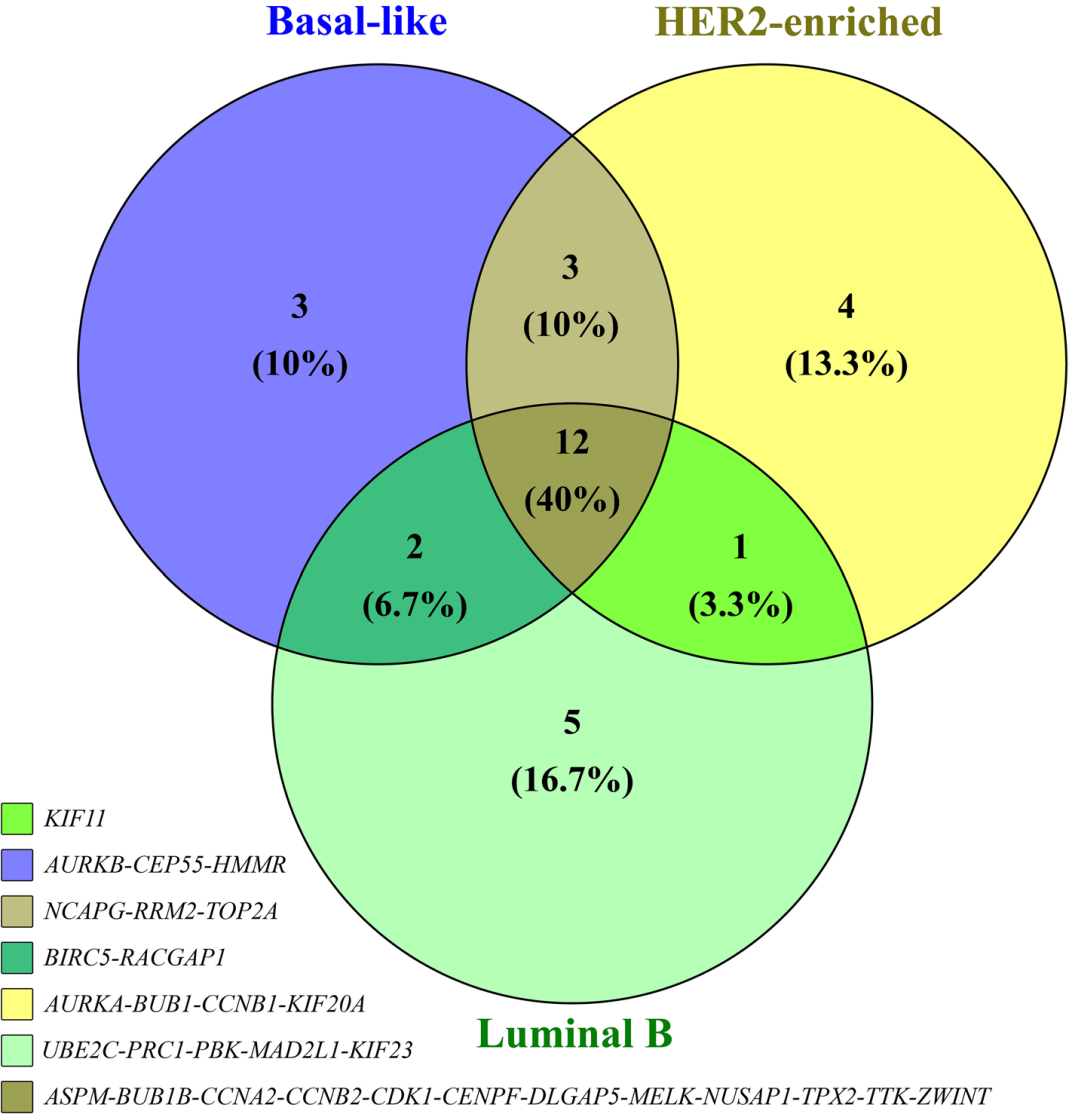
The overlap of upregulated hub genes among Luminal B, HER2-enriched, and Basal-like breast cancer.

To investigate the possible role of the hub-upregulated genes that were identified as essential drivers in both primary and bone-metastatic breast cancer in various subtypes of breast cancer, different PAM50 subtypes of breast cancer were compared to the healthy samples (Volcano plot and UMAP diagram of samples’ separation are available in Supplementary 7). After eliciting the PPI network for each group, the top 20 hub upregulated genes were highlighted using the Cytohubba plug-in. Then, the common hub upregulated genes among Luminal B, Basal-like, and HER2-enriched breast cancer were discerned by the Venny tool, as shown in Fig. 12. In the final step, these genes were compared to the common hub upregulated genes between primary and bone-metastatic breast cancer to discover any consistent pattern. Three (*CDK1*, *TTK*, and *MELK*) of 12 common upregulated hub genes among Luminal B, Basal-like, and HER2-enriched were also observed in common upregulated hub genes between primary and bone-metastatic breast cancer. Moreover, it was found that some of the other genes were also shared between one particular type of breast cancer and this study's hub gene. For instance, while *AURKB* was identified as one of the hub genes in Basal-like breast cancer, *AURKA* was recognized as a critical gene in HER2-enriched breast cancer.

These findings show the significance of previously-identified kinases, including *AURKB*, *MELK*, *TTK*, and *KIF20A,* in the occurrence of various subtypes of breast cancer and even its progression toward the metastatic phase.

### Bone-metastatic and skin-metastatic breast cancer: similarities and differences

**Figure 13:**
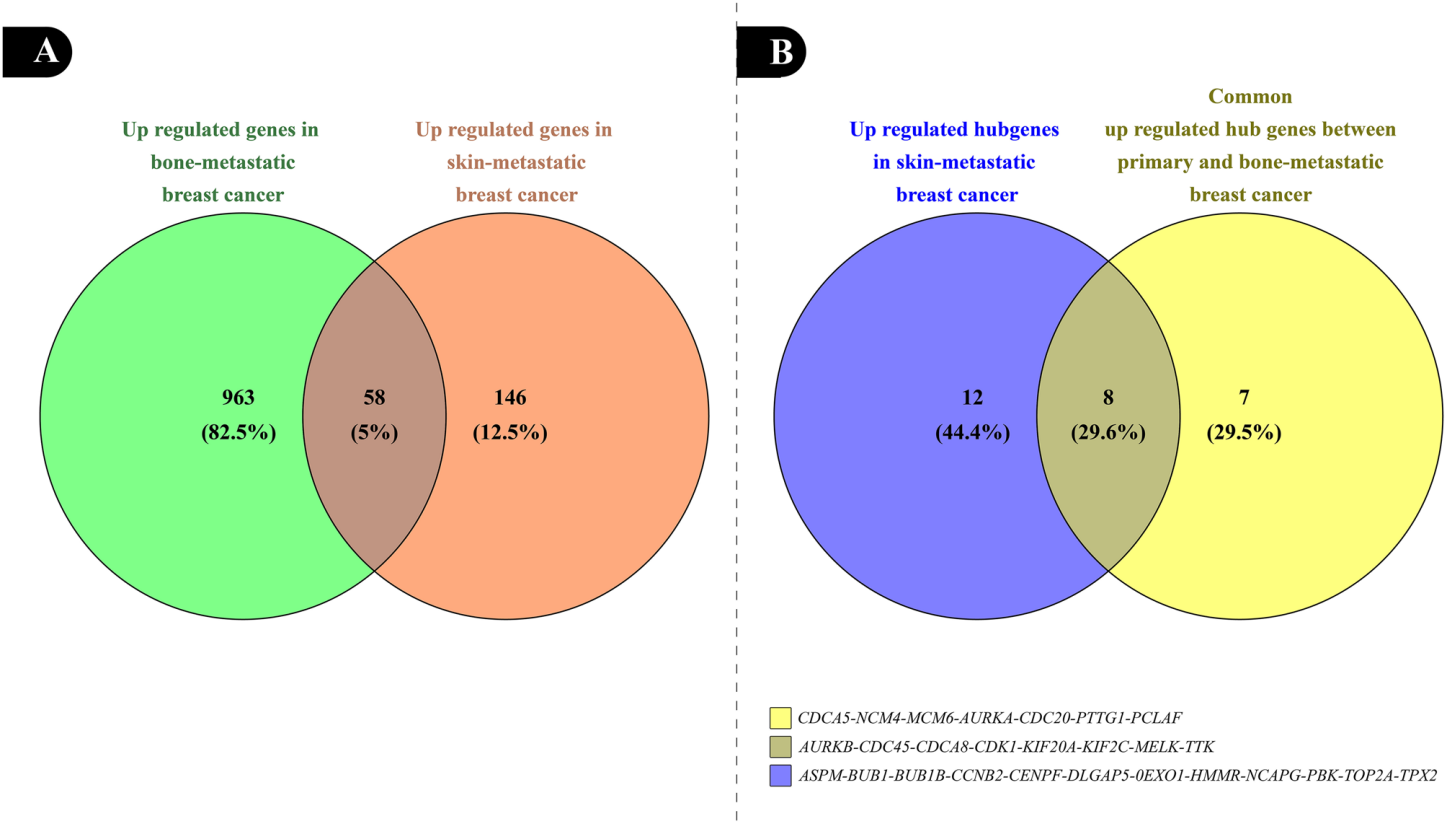
The Venn diagram of upregulated genes in the skin- and bone-metastatic breast cancer tumors (**A**); and hub genes in skin-metastatic versus common hub genes in primary and bone-metastatic breast cancer (**B**).

As described before, appropriate samples of GSE56493 were selected to identify significant DEGs in skin-metastatic breast cancer. While the comparison between upregulated genes in skin-metastatic and bone-metastatic breast cancer revealed slight overlap among these two types of metastasis (overlap percentage: 5%), the overlap percentage jumped to 29.6% when the hub genes of both groups were compared. Figure 13 displays the Venn Diagram of this comparison schematically.

Once again, this analysis emphasized the importance of suggested upregulated hub genes as novel targets for drug discovery, inhibitor designing, and siRNA-based therapeutic agents. It also indicated that any potential inhibitor against the introduced hub genes in this study could target not only various types of primary breast cancer but also its possible metastasis to bene and skin vigorously.

## Discussion

Nowadays, breast cancer is regarded to be one of the most critical health and medical issues due to its high prevalence among females worldwide. Epidemiological studies have suggested that in the United States, breast cancer holds the second place among all various diseases, accidents, etc., resulting in women’s death^74^. The high prevalence of breast cancer, the emergence of drug-resistant cancerous cells, and the possibility of metastasis, which causes the involvement of other organs, prove the urgent demand for apprehending the molecular basis of this disease^75^. Herein, the differential expresses genes in the breast cancer primary tumor cells and P7731 cell line (bone metastasis for breast cancer) was probed to detect the common DEGs between these cells to shed light on designing novel therapeutic agents targeting both of them efficiently.

To dive into details, the gene ontology analysis demonstrates that common upregulated genes are enriched in the crucial biological process, including the DNA-related pathways such as DNA replication and DNA metabolic process confirming the significance of such genes in cancer occurrence and cell proliferation. Similarly, previous studies have shown a strong relationship between gene ontology and mammary malignancies^76^. Furthermore, from a molecular function perspective, DNA replication, cadherin, and mRNA binding functions were the most relevant MFs to upregulated genes. The role of deregulation of cadherin and catenin in cancer progression, despite their primary role in mammary development, was investigated by Pamela Cowin et al.^77^. In addition, it is worth mentioning that although a low percentage of genes contribute to intracellular bounded and non-bounded organelles and nucleus-related components, these cellular components have the most robust connection with common upregulated genes. Investigating the gene ontology, particularly cellular components, is vital since their role in breast cancer pathways has been observed and proven repeatedly^78,79^. The analysis of common upregulated genes between the defined groups revealed that most of the top 10 pathways having the most meaningful association (based on Log adjusted *p*-value) are related to the cell cycle, apoptosis, and cancer-related pathways. For instance, 17.74% of 573 common upregulated genes that play critical roles in the cell cycle pathways have the most meaningful correlation with both primary and metastatic breast cancer types. The significance of cell cycle pathways was also investigated in other research designs^80^, highlighting the importance of the result of the present study.

Moreover, the obtained results also showed that some influential pathways in cancer (e.g., mRNA surveillance) are highly associated with the common downregulated genes. Thus, it is safe to say that such genes dangerously impact cell proliferation. The high complexity of the PPI network of common DEGs constructed using the STRING database and Cytoscape application indicated the importance of genes contributing to both primary and metastatic cancer types. Notably, the PPI analysis is noticeable since other studies have proven their functions in breast cancer^72^ and their relation with other types of cancers, such as colon cancer^73^.

After the detection of hub genes utilizing the PPI network of common upregulated genes, the top five hub genes, including *AURKA*, *AURKB*, *KIF20A*, *MELK,* and *TTK* that played the most critical role in both primary tumor cell and P7731 cell line, were selected to validate the study. Additionally, the investigation of these five genes by Human Atlas Protein, GEPIA, and Kaplan–Meier databases showed their correlation with tumorigenesis, so it validated the results. Aurora kinase A (*AURKA*) and Aurora kinase B (*AURKA*), which their overexpression is highly related to cancer emergence, have been found to play a crucial role in cell proliferation and division^81,82^. AURKA or STK61, a protein from the serine/threonine kinases family, is essential for the cell division during mitosis. It is also highlighted as one of the essential biomarkers in cancer prognosis, which its overexpression may activate deleterious phosphorylation pathways and induce cancer^83^. Moreover, *AURKA* has also a robust correlation with other genes contributing to Wnt and Ras-MAPK signaling pathways^84^. Due to the cogent effect of *AURKA* in various cancers, different inhibitors are designed and tested to halt cancer progress by suppressing this gene’s upregulation^85,86^. AURKB, which was detected as the second pivotal hub gene in this study, also belongs to serine/threonine kinases, and its amplification is clarified to result in tumorigenesis in diverse organs^87^. In fact, *AURKB* is proven to ameliorate the cell cycle by targeting different genes contributing to mitosis. To be more precise, *AURKB* diminishes the expression of p21 by inhibiting p53 activity; thereby, causing upregulation of CDK1, eventually leading to cell division and increasing the tumor cell survival^88^. *KIF20A* gene encodes a protein named as kinesin family member 20A, which is necessary for the spindle assembly and chromosome segregation during mitosis, particularly anaphase and cytokinesis^89^. This gene is also highly associated with other crucial genes in cell proliferation (e.g., *MKLP1*, *PLK1*, and *RAB6*). Furthermore, *CDK1* is also affected by the proportion of *KIF20A* overexpression, which highlights the decisive role of *KIF20A* in the cell division and mitosis^89^. It is indicated that other types of cancer, like bladder cancer, are also caused by the upregulation of this gene resulting in more complicated tumor differentiation and a lower rate of survival in patients^90^.

The present results pronounce the importance of the Maternal embryonic leucine zipper kinase (*MELK*) gene as one of the most influential hub genes in both primary tumor cells and the P7731 cell line. More specifically, *MELK* is also a kinase that noticeably exerts its oncogenic impacts by interacting with cyclin B and cyclin D1 genes^91^. This gene is related to tumors’ aggressive growth and drug resistance emergence in cancerous cells^92^. In addition, it is acknowledged that inhibiting *MELK*, as a pivotal gene in breast cancer, can reduce cell division by suppressing the expression of cyclin B and D1^70^ and increasing the sensitivity of breast cancer tumors to chemo- and radiotherapy^92^. Threonine and tyrosine kinase (*TTK*), the last important hub gene utilized to validate the outcomes of the present study, is part of the spindle assembly checkpoint and has an essential role in the chromosomal separation during mitosis^93^. In addition, the meaningful relation of *TTK* (which is known as a biomarker for the poor prognosis of various cancer, including breast^94^) with tumorigenesis, especially the advent of aneuploidy tumors, is already discovered^95^.

Various proteins and genes primarily having crucial roles in cell division are already mentioned as other important hub genes in multiple types and metastasis of breast cancer. On the other hand, uncontrolled cell division is an undeniable part of the “cancer” definition^96^. While CDC45 is indirectly required for the initiation of DNA replication due to its high connections with other cell proliferation genes like MCMs^97^, CDC20 directly activates anaphase-promoting complex (APC/C), resulting in chromatid separation and cell going through anaphase in mitosis^98^. Moreover, due to being one of the hub genes in bioinformatics analysis by Arulprakasam Ajucarmelprecilla et al*.*^99^, CDC45 is reportedly a crucial biomarker for gastric cancer as well. CDCA8 and CDCA5 are the other stated hub genes that regulate the cell division cycle by coding an essential protein complex for chromosomal migration called chromosome passenger complex (CPC)^100^ and chromatid cohesion in mitosis stabilizing^101^, respectively. Similarly, *KIF20A* is also involved in chromosomal transportation by CPC during mitosis^102^. As the role of other hub genes including *MELK*, *AURKA* and *AURKB* genes are explained before, it can be concluded that suppressing some of the pivotal genes (not all of them) in the cell division can efficiently pause the whole process of tumorigenesis and cell proliferation. Although most of the important genes in tumorigenesis seems to be explicitly liked to cell cycle, some other enzymes and proteins like *TYMS* and *FN1* are also reported to be effective in breast adenocarcinoma by Jhansi Pandi et al.^103^. Finally, suppression of the genes that are statistically ranked as the more important genes is expected to be more effective in cancer treatment; however, the clinical research may be accompanied by other reasons due to the unknown biological functions and metabolisms in the cell.

The analysis of genes’ transcription in other molecular subtypes of primary breast cancer, including basal-like (the closest PAM50 subtype to triple-negative breast cancer), HER2-enriched, and Luminal B, proved the significance of the mentioned above hub genes. Moreover, some genes, such as *TTK* and *MELK,* were repeatedly observed in various PAM50 subtypes of primary mammary carcinoma and even in bone-metastatic and skin-metastatic breast cancer. Also, *AURKB* and *KIF20A*, the other two genes amongst five critical genes which are introduced as pivotal targets for prospective drug discovery and inhibitor designing, were indicated to play a crucial role in breast carcinoma metastasis to the skin. All in all, it appears that *AURKB*, *AURKA*, *TTK*, *MELK,* and *KIF20A* initiate the signaling pathways that eventually result in uncontrolled cell proliferation; therefore, inactivation and degradation of such genes’ proteins or mRNA illuminates a novel strategy to target the cancerous cells efficiently^92,104,105^. Based on the statistical analyses of the PPI network of upregulated genes, *AURKA*, *AURKB*, *TTK*, *MELK*, and *KIF20A* were also involved in module 1, indicating their importance both in primary tumorigenesis and cancer progression.

As pointed before, modules are defined as a group of highly-related genes in a gene regulatory (GR) or PPI network primarily affected by the same transcription factors^106^. Since the same TFs play an essential role in the transcription/expression of co-expressed genes, simultaneous transcription is usually observed for the genes that are categorized as one module in a GR or PPI network. In other words, modules are composed of a highly connected cluster of genes forming a subgraph in the leading network; such genes are involved in the same biological pathway or function, and targeting one may interrupt the whole module and even the entire network^107^. Clustering plug-ins such as MCODE follow a statistical approach to identify and rank the most critical modules in a PPI using mathematical parameters like K-Core. The top 3 modules usually reveal the vital genes involved in tumorigenesis and often include the hub genes of a particular network, increasing the validity of previous calculations. As can be seen, most of the 15 upregulated hub genes are also present in the module 1 which emphasizes their role in cancer-relevant pathways.

Five various databases to verify the trueness of the study hypothesis, analysis conduction, and integrity of detected hub genes indicated that the upregulation of detected common hub genes is already recorded in breast cancer patients. Also, it shows that the overexpression of upregulated hub genes causes tissue deformation, and it is correlated with less probability of survival in people diagnosed with breast carcinoma. On the other hand, the drawn heat map demonstrated that most of the marked genes are increasingly vital for breast cancer cell lines authorizing the results once again. The Kaplan–Meier analysis was selected as one of the validating approaches for detected hub genes in the PPI network of common upregulated genes showing a significant Hazard Ratio (HR). The HR is one of the most-used statistical parameters in clinical trials or survival analysis, indicating the possibility of a particular event like death/survival in two identical groups (test group vs. control group) which only have one distinct characteristic such as a specific expression of a gene over a period of time (month/year)^108^. As there is a downward slope in the survival of cancer patients, as displayed in Kaplan–Meier curves, HR determines the probability of death in the patients who have shown the higher expression rate for the inquired gene^109^. GEPIA analysis disclosed that breast cancer patients have significantly higher expression of *AURKA* (4.0 vs. 1.5), *AURKB* (4 vs. 1), *TTK* (2.5 vs. 0.5), *MELK* (3.0 vs. 0.5), *KIF20A* (3.5 vs. 0.5) genes compared to healthy people. On the other hand, Kaplan–Meier curves revealed the HR value to be 1.87, 1.43, 1.87, 1.81, 1.66 for *AURKA*, *AURKB*, *TTK*, *MELK*, and *KIF20A*, respectively, meaning the risk of death to be about 87%, 43%, 87%, 81% and 66% higher in patients whom these genes are upregulated in comparison to the patients with regular expression. To conclude, the conducted analyses showed that detected hub genes in this study are pretty suitable targets for future drug discovery research as they are vital not only in the various subtypes of primary breast carcinoma but also in both bone- and skin-metastatic mammary cancer.

Although there have been some recent endeavors for designing effective drugs against introduced hub genes in this study, more efficient research is required due to unmet achievements in this area. As the *MELK* gene is discovered to be responsible for Glioblastoma multiforme (GBM), OTSSP167, a MELK inhibitor recently in clinical trial phase I/II, has been synthesized for cancer treatment through MELK inhibition^110^. OTSSP167, an oral inhibitor of *MELK*, effectively speeds up the destabilization of MELK by stopping its autophosphorylation, which is necessarily demanded for MELK protein stability and function^111,112^. MELK-8a is another empirically used substance that has effectively shown selective inhibition of the MELK protein^113^. The valuable attempts for detecting and targeting the important genes in gastroenteropancreatic neuroendocrine tumors (GEP-NETs)^114^, prostate cancer^115^, etc., provided new inhibitors for Aurora genes (i.e., *AURKA* and *AURKB*) like ZM447439 and Hesperadin ultimately. Aurora inhibitors target the AURKB protein by acting as an ATP-competitive agent and suppressing DNA-related mechanisms^116^. While Hesperadin is typically used at a concentration of approximately 50–500 nM^117^, ZM447439 is administered around 2–20 μM^118^, determining the higher potency of Hesperadin for *AURKB* inhibition. Furthermore, Tozasertib (VX-680) and LY3295668 are other Aurora inhibitors actively targeting *AURKA*^119^. Since immature hematopoietic cells have proved to express KIF20A highly, there has been enormous research to find KIF20A to treat leukemia. DIACC2010 is a KIF20A inhibitor in the preclinical phase, which has passed in vitro step successfully with a median half inhibitory concentration (IC50) of 40 nM^120^. Due to the high importance of *TTK* in hepatocellular carcinoma (HCC), CFI-402257 has been designed to knock out the *TTK* gene^121^. As stated before, there are recently some other therapeutic agents for various cancers’ treatment and critical genes inhibition; however, more in silico, in vitro, and clinical trials are required for accurate tumor targeting.

To sum up, all of the abovementioned genes validated the results and were identified as the most critical hub genes in both primary and bone metastatic cancer cells. To extend the deduced results of this research and synthesize more accurate inhibitors or other therapeutic agents, other high throughput methods such as RNA-seq as well as other metastatic tumors can be used as models.

## Conclusion

In conclusion, the most critical differentially expressed genes were detected by utilizing a comprehensive transcriptomic analysis. The usage of bioinformatics tolls resulted in the identification of *AURKA*, *AURKB*, *TTK*, *MELK* and *KIF20A* as top 5 hub genes involved in breast cancer occurrence and progression. Other bioinformatics databases and software were used to reveal the characterization of identified genes. Since we authorized the integrity of determined hub DEGs, it is now proven that such genes can now be counted as novel targets for breast carcinoma treatment by designing new drugs, inhibitors, and siRNA-based therapeutics. Furthermore, the high expression level of identified genes has made them advent biomarkers for mammary carcinoma diagnosis by simple evaluating methods. Moreover, the efficiency of cell lines as emerging cancer models is approved in this study once again. Although the significance of found hub genes in the occurrence and progression of diverse types of breast cancer from primary to advanced-metastatic stages was validated by applying various experimental-clinical databases, the importance of further experiments should not be ignored. As some other genes such as *BUB1* and *NCAPG* were also reported to be influential in breast adenocarcinoma using bioinformatics tool^103^, it appears that a large-scale clinical research is highly required for detection of most critical genes. Ultimately, this study provides a robust basis to discover and highlight undisclosed signaling pathways in breast cancer by marking new genes, TFs, metabolites, kinases, miRNAs, and PP interactions.

## Supplementary Information

Supplementary Information 1.

Supplementary Information 2.

Supplementary Information 3.

Supplementary Information 4.

Supplementary Information 5.

Supplementary Information 6.

Supplementary Information 7.

## Data Availability

All data are available in the text of the article and Supplementary files. All data used an analyzed in this article are freely available in the mentioned databases e.g. Gene Expression Omnibus (https://www.ncbi.nlm.nih.gov/geo/).

## Abbreviations

RAG-2: Recombination activating gene 2
CDK4/6: Cyclin-dependent kinase 4 and 6
BRCA-2: Breast cancer gene type 2
EGFR: Epidermal growth factor receptor
DEG: Differentially expressed gene
GEO: Gene expression omnibus
ChEA: ChIP enrichment analysis
FDR: False discovery rate
BP: Biological process
CC: Cellular component
miRNA: Micro-ribonucleic acid
NGS: Next generation sequencing
PPI: Protein–protein interaction
MCC: Maximal clique centrality
MAPK14: Mitogen-activated protein kinase-14
CK2ALPHA: Casein kinase two alpha-1
CDC2: Cyclin-dependent kinase 2
AURKB: Aurora kinase B
TTK: Tyrosine-threonine kinase
GRN: Gene regulatory network
FN1: Fibronectin 1
NCAPG: Non-SMC condensin I complex subunit G
BUB1: Serine/threonine protein kinase
TNBC: Triple-negative breast cancer
GEP-NET: Gastro-entero-pancreatic neuroendocrine tumors
HCC: Hepatocellular carcinoma
CPC: Chromosome passenger complex
IDC: Invasive ductal carcinoma
BRCA-1: Breast cancer gene type 1
HER2: Human epidermal growth factor receptor 2
Fc: Fold change
NCBI: National center for biotechnology information
TRE: Trans regulatory elements
TF: Transcription factor
GO: Gene ontology
MF: Molecular function
KEGG: Kyoto encyclopedia of genes and genomes
MTI: Micro RNA-target interaction
HMDB: Human metabolites database
MCODE: Molecular complex detection
DNA-PK: DNA-dependent protein kinase
CCL: Cancer cell line
AURKA: Aurora kinase A
MELK: Maternal embryonic leucine zipper kinase
*P-*value: Probability value
IC50: Half inhibitory concentration
TYMS: Thymidylate synthetase
HR: Hazard ratio
GBM: Glioblastoma multiforme
APC/C: Anaphase-promoting complex
NP: Nanoparticle
