# Supplementary material for: Identification, characterization, and prognosis investigation of pivotal genes shared in different stages of breast cancer
Source: Sci Rep. 2023 May 25;13:8447. doi: 10.1038/s41598-023-35318-x (PMC10212935; doi:10.1038/s41598-023-35318-x)
Supplement: Supplementary file 1 — Supplementary Information 1. [file 41598_2023_35318_MOESM1_ESM.docx]

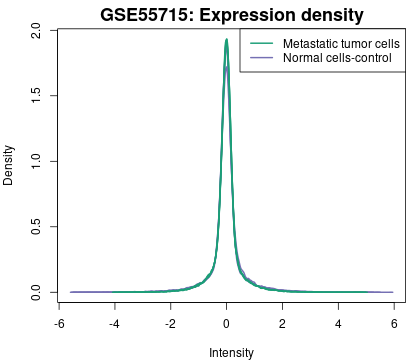
Supplementary 1


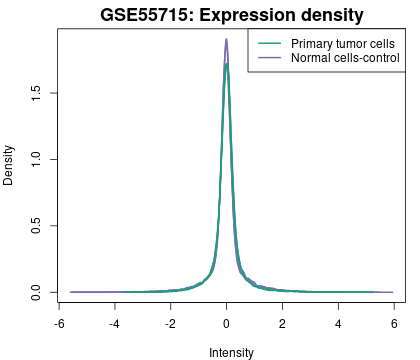


1. Normal distribution of defined groups


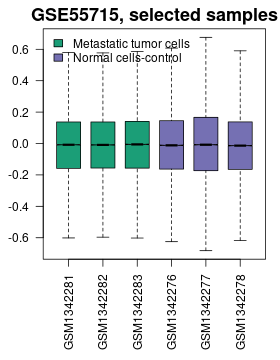

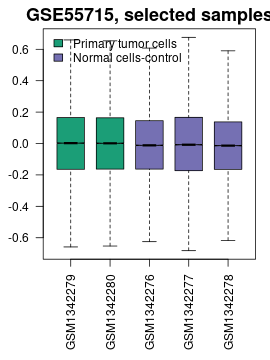


1.
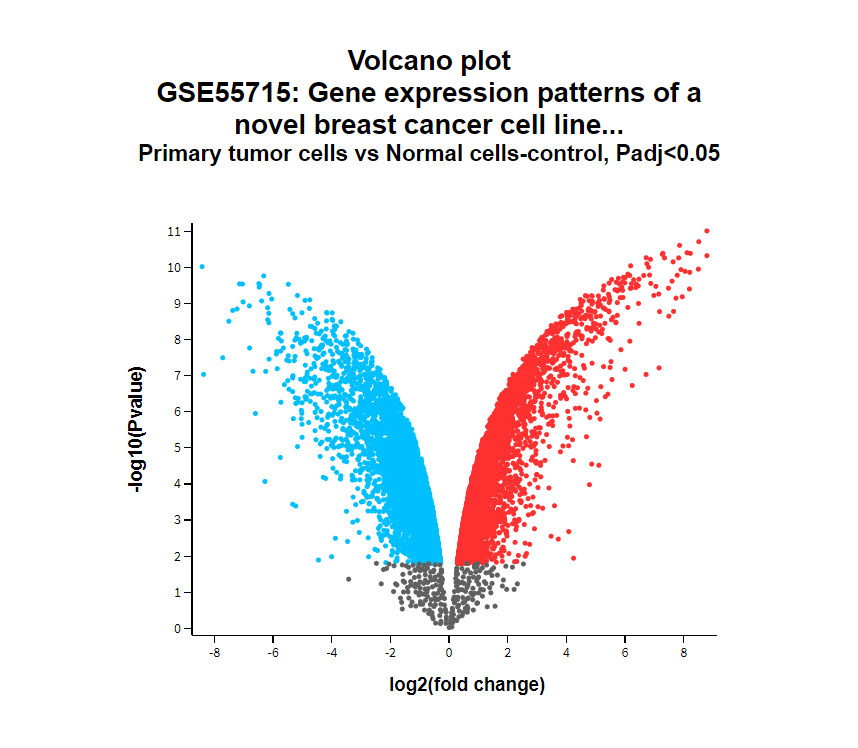
Plot box of defined groups


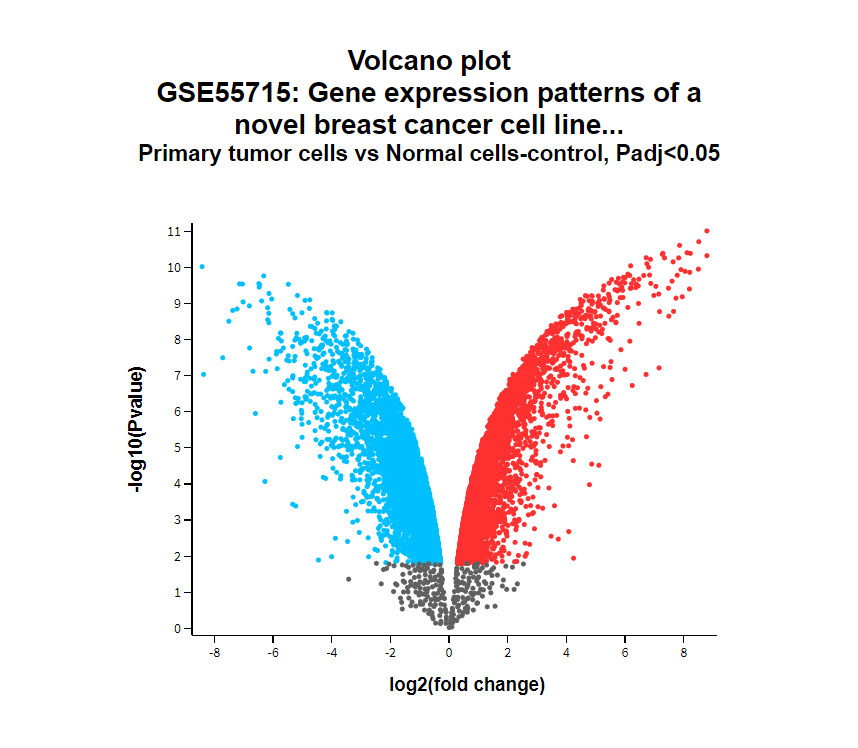

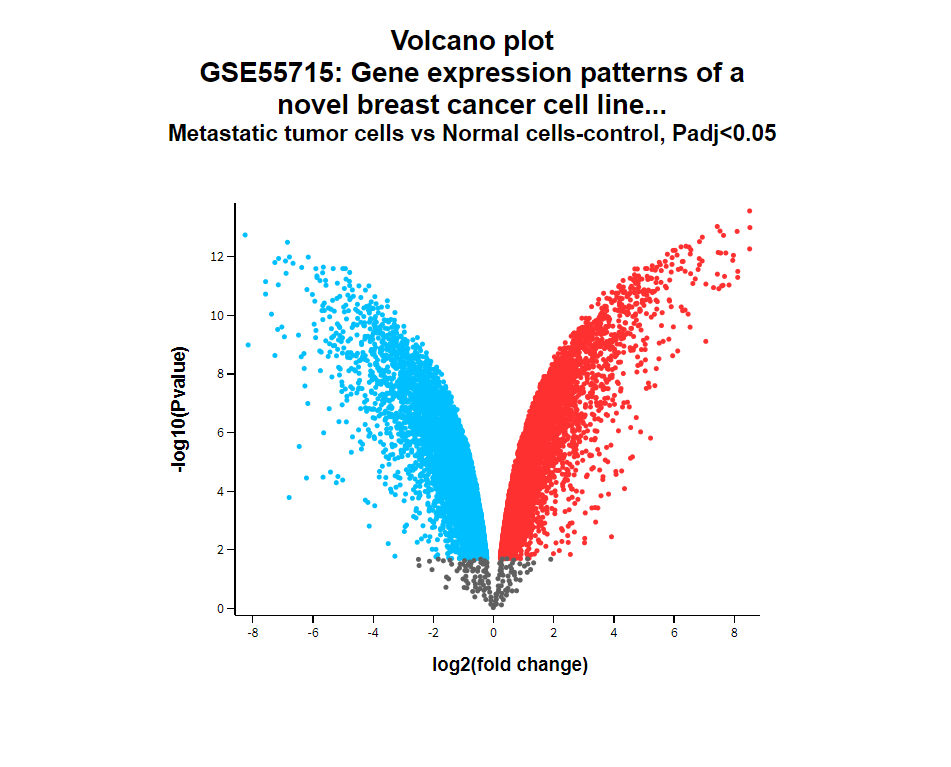


1. Volcano plots of 2 sorted groups
