# Supplementary figures and images for: Identification, characterization, and prognosis investigation of pivotal genes shared in different stages of breast cancer
Source: Sci Rep. 2023 May 25;13:8447. doi: 10.1038/s41598-023-35318-x (PMC10212935; doi:10.1038/s41598-023-35318-x)

Supplementary 2


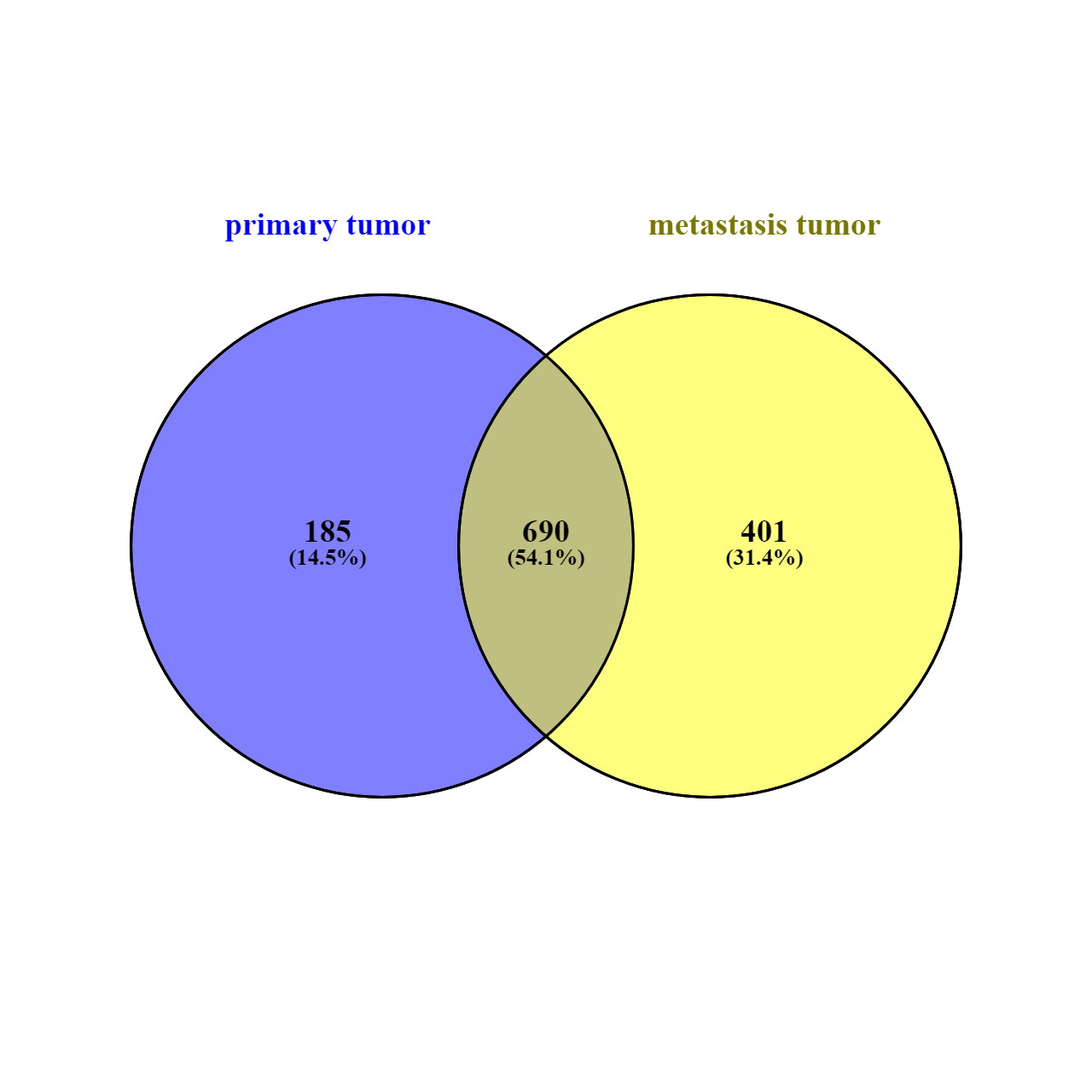


Downregulated


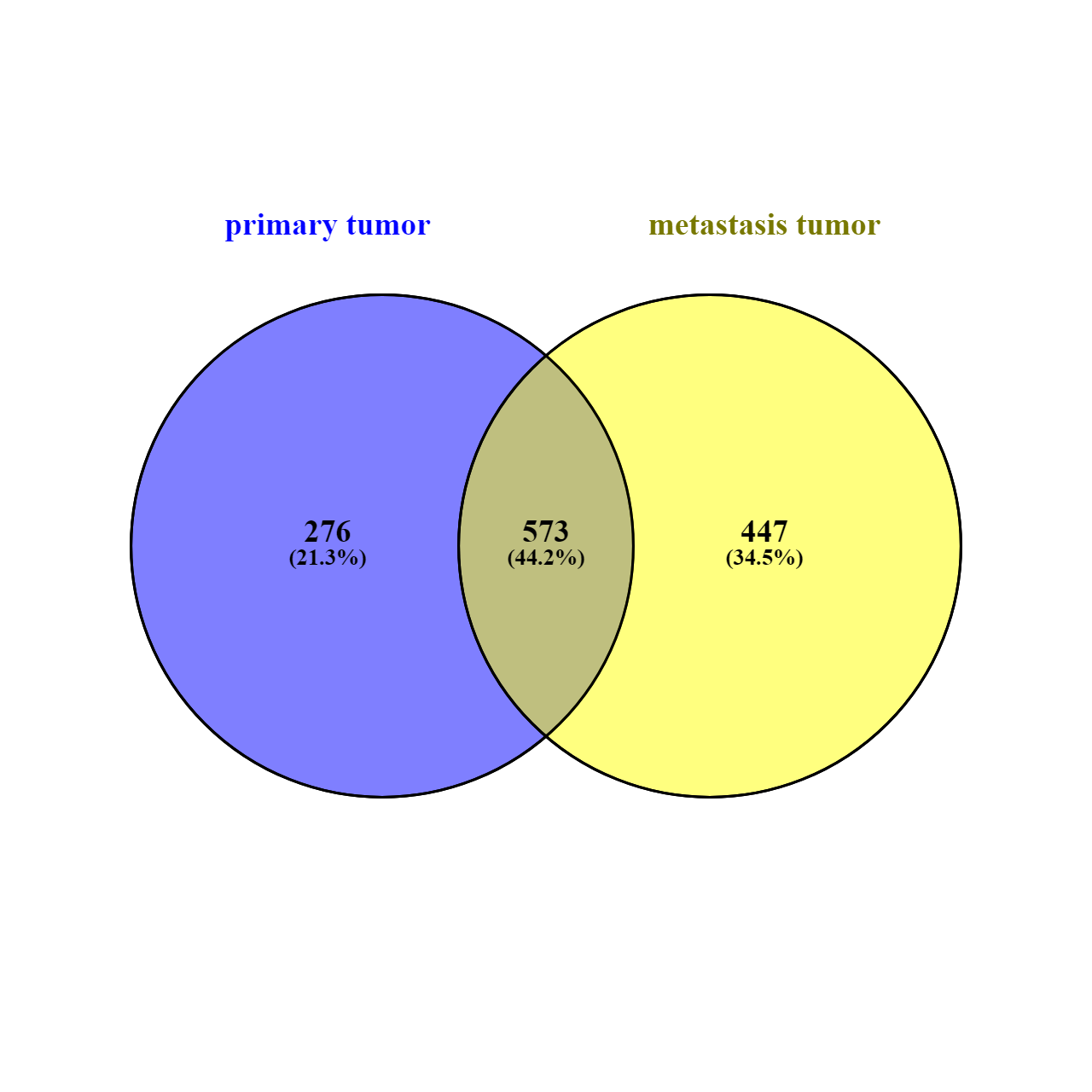


Upregulated

Supplement: Supplementary file 2 — Supplementary Information 2. [file 41598_2023_35318_MOESM2_ESM.docx]

**Supplementary 7**


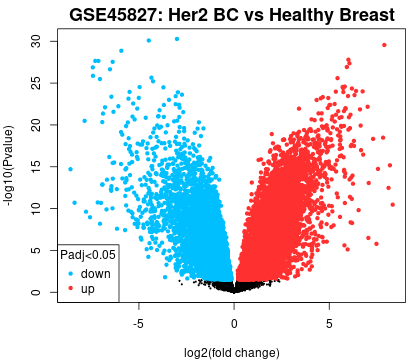


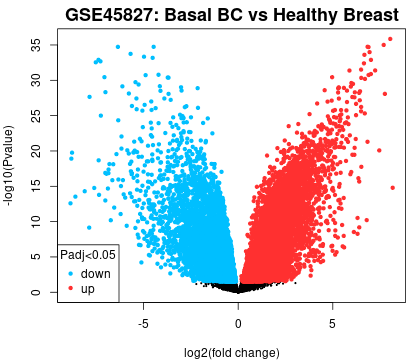


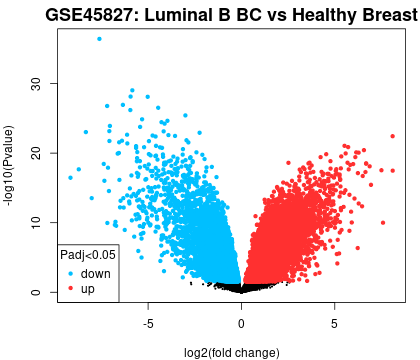


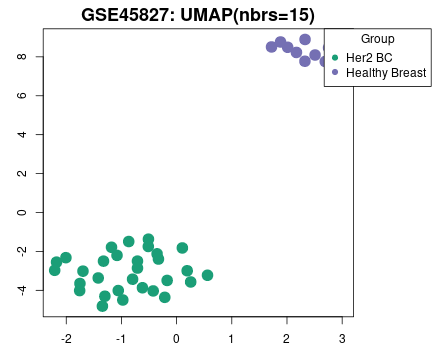


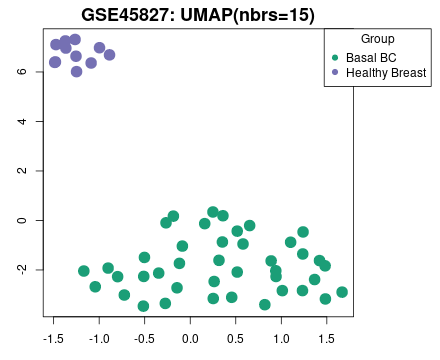


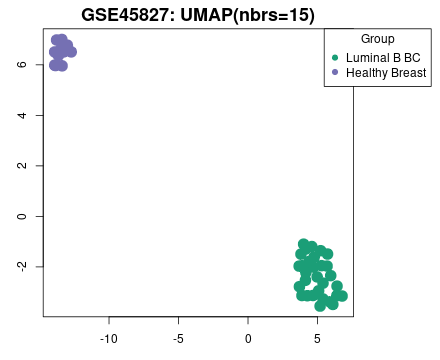

Supplement: Supplementary file 7 — Supplementary Information 7. [file 41598_2023_35318_MOESM7_ESM.docx]
