# Supplementary material for: Identification, characterization, and prognosis investigation of pivotal genes shared in different stages of breast cancer
Source: Sci Rep. 2023 May 25;13:8447. doi: 10.1038/s41598-023-35318-x (PMC10212935; doi:10.1038/s41598-023-35318-x)
Supplement: Supplementary file 3 — Supplementary Information 3. [file 41598_2023_35318_MOESM3_ESM.docx]

| Common upregulated transcription factors’ gene symbol | | | | | |
| --- | --- | --- | --- | --- | --- |
| AIRE | ATF4 | CBX2 | CREB1 | CUX1 | E2F2 |
| FOXK1 | FOXN3 | HOXB7 | HOXC6 | JUN | JUNB |
| MZF1 | NACC2 | PITX1 | PRMT3 | PRRX1 | PRRX2 |
| RARA | REPIN1 | SP3 | STAT1 | TCF20 | TEAD4 |
| TIGD5 | ZNF146 | ZNF302 | ZNF32 | ZNF394 | ZNF430 |
| ZNF443 | ZNF473 | ZNF549 | ZNF577 | ZNF598 | ZNF652 |
| ZNF69 |  | | | | |

Supplementary 3

| Common downregulated transcription factors’ gene symbol | | | | | | |
| --- | --- | --- | --- | --- | --- | --- |
| CGGBP1 | ELF2 | ELF5 | ETV5 | GPBP1 | GRHL1 | HOMEZ |
| HOXA5 | KDM2B | KLF5 | LTF | MBNL2 | MYC | MYNN |
| NFAT5 | NFIC | NME2 | PURA | SOX10 | SP110 | SP3 |
| SREBF2 | TFAP2B | TFCP2L1 | THYN1 | TTF1 | USF2 | YY1 |
| ZBTB16 | ZBTB44 | ZHX1 | ZNF14 | ZNF140 | ZNF180 | ZNF521 |
| ZNF544 | ZNF569 | ZNF664 | ZNF766 | ZNF91 | ZSCAN31 |  |
