# Supplementary material for: Identification, characterization, and prognosis investigation of pivotal genes shared in different stages of breast cancer
Source: Sci Rep. 2023 May 25;13:8447. doi: 10.1038/s41598-023-35318-x (PMC10212935; doi:10.1038/s41598-023-35318-x)
Supplement: Supplementary file 5 — Supplementary Information 5. [file 41598_2023_35318_MOESM5_ESM.docx]

Supplementary 5
